# MicroRNAs Associated with Parenchymal Hematoma After Endovascular Mechanical Reperfusion for Acute Ischemic Stroke in Rats

**DOI:** 10.3390/biomedicines13020449

**Published:** 2025-02-12

**Authors:** Jin-Kun Zhuang, Zhong-Run Huang, Wang Qin, Chang-Luo Li, Qi Li, Chun Xiang, Yong-Hua Tuo, Zhong Liu, Qian-Yu Chen, Zhong-Song Shi

**Affiliations:** 1 Department of Neurosurgery, Sun Yat-Sen Memorial Hospital, Sun Yat-Sen University, Guangzhou 510120, China; zhuangjk@mail2.sysu.edu.cn (J.-K.Z.); huangzhr3@mail2.sysu.edu.cn (Z.-R.H.); qinw26@mail2.sysu.edu.cn (W.Q.); lichluo@mail3.sysu.edu.cn (C.-L.L.); liqi85@mail2.sysu.edu.cn (Q.L.); xiangch7@mail2.sysu.edu.cn (C.X.); 2 RNA Biomedical Institute, Sun Yat-Sen Memorial Hospital, Sun Yat-Sen University, Guangzhou 510120, China; chenqycherry@126.com; 3 Nanhai Translational Innovation Center of Precision Immunology, Sun Yat-Sen Memorial Hospital, Sun Yat-Sen University, Foshan 528208, China; 4 Department of Neurosurgery, The First Affiliated Hospital, Sun Yat-Sen University, Guangzhou 510080, China; tyh2150@126.com (Y.-H.T.); liuzh_neurosurgery@163.com (Z.L.); 5 Department of Neurosurgery, The Second Affiliated Hospital, Guangzhou Medical University, Guangzhou 510260, China; 6 Department of Neurosurgery, Zhongshan Hospital of Xiamen University, School of Medicine, Xiamen University, Xiamen 361005, China; 7 Guangdong Province Key Laboratory of Brain Function and Disease, Sun Yat-Sen University, Guangzhou 510080, China

**Keywords:** acute ischemic stroke, endovascular treatment, hemorrhagic transformation, parenchymal hematoma, oxygen–glucose deprivation/reoxygenation, microRNAs

## Abstract

**Background/Objectives:** Hemorrhagic transformation after endovascular thrombectomy predicts poor outcomes in acute ischemic stroke with large-vessel occlusion. The roles of microRNAs (miRNAs) in the pathogenesis of parenchymal hematoma (PH) after endovascular thrombectomy still remain unclear. This study aimed to investigate the miRNA and mRNA regulatory network associated with PH after mechanical reperfusion in an animal stroke model and an oxygen–glucose deprivation/reoxygenation (OGD/R) model. **Methods:** Twenty-five miRNAs were assessed in a mechanical reperfusion-induced hemorrhage transformation model in rats under hyperglycemic conditions receiving 5 h middle cerebral artery occlusion. The differentially expressed miRNAs associated with PH were assessed in a neuron, astrocyte, microglia, brain microvascular endothelial cell (BMEC), and pericyte model of OGD/R. The predicted target genes of the differentially expressed miRNAs were further assessed in the animal model. The miRNA-mRNA regulatory network of PH was established. **Results:** Thirteen down-regulated miRNAs (miRNA-29a-5p, miRNA-29c-3p, miRNA-126a-5p, miRNA-132-3p, miRNA-136-3p, miRNA-142-3p, miRNA-153-5p, miRNA-218a-5p, miRNA-219a-2-3p, miRNA-369-5p, miRNA-376a-5p, miRNA-376b-5p, and miRNA-383-5p) and one up-regulated miRNA (miRNA-195-3p) were found in the rat peri-infarct with PH after mechanical reperfusion. Of these 14 PH-related miRNAs, 10 were significantly differentially expressed in at least two of the five neuron, astrocyte, microglia, BMEC, and pericyte models after OGD/R, consistent with the animal stroke model results. Thirty-one predicted hub target genes were significantly differentially expressed in the rat peri-infarct with PH after mechanical reperfusion. Forty-nine miRNA-mRNA regulatory axes of PH were revealed, and they were related to the mechanisms of inflammation, immunity, oxidative stress, and apoptosis. **Conclusions:** Fourteen miRNAs were associated with PH after mechanical reperfusion in the rat stroke and the OGD/R models. Simultaneously differentially expressed miRNAs and related genes in several cells of the neurovascular unit may serve as valuable targets for PH after endovascular thrombectomy in acute ischemic stroke.

## 1. Introduction

Endovascular thrombectomy (EVT) alone or intravenous alteplase plus EVT can provide clinical benefit in patients with acute ischemic stroke (AIS) due to large-vessel occlusion, as shown in randomized controlled trials [1,2,3,4,5,6]. Half of patients with AIS receiving EVT have favorable outcomes. Hemorrhagic transformation (HT) occurs in one-third of patients with AIS receiving EVT alone or intravenous alteplase plus EVT, even in half of the patients receiving EVT 6 h after stroke [7,8,9]. Reductions in symptomatic HT, one of the predictors of poor outcomes, have recently been observed, accompanied by the advancement of EVT. However, parenchymal hematoma (PH) is still associated with functional dependence after EVT [10,11,12].

Blood–brain barrier (BBB) dysfunction is vital in cerebral ischemia–reperfusion injury and HT [13,14,15]. Early BBB disruption predicts HT and poor outcomes in AIS patients receiving EVT [16,17,18]. Non-coding RNAs can contribute to cerebral ischemia–reperfusion injury and act as non-invasive biomarkers for the diagnosis and prognosis of HT in AIS patients. MicroRNAs (miRNAs) regulate gene expression post-transcriptionally, playing crucial roles in ischemic stroke pathology. By modulating inflammation, oxidative stress, apoptosis, and BBB integrity, miRNAs act as key molecular mediators in ischemia–reperfusion injury [19,20,21,22]. PH represents a severe complication of ischemic stroke, leading to worsened clinical outcomes. Hematoma growth post-reperfusion is driven by BBB disruption, increased vascular permeability, and exacerbated inflammatory responses. In previous studies, we obtained preliminary results of the miRNA expression profile in rat ischemic hemispheres with HT after mechanical reperfusion using a miRNA array. A nicotinamide adenine dinucleotide phosphate oxidase inhibitor reduced HT after endovascular mechanical reperfusion and altered the levels of several miRNAs [23,24]. However, the molecular pathways contributing to PH after mechanical reperfusion remain poorly defined. In particular, the specific miRNA–mRNA regulatory network underlying PH has not been comprehensively studied. Previous studies have focused on miRNAs as diagnostic and prognostic biomarkers for ischemic stroke and HT but have mainly overlooked their role in the hyperglycemia-exacerbated environment associated with PH.

**Figure 1:**
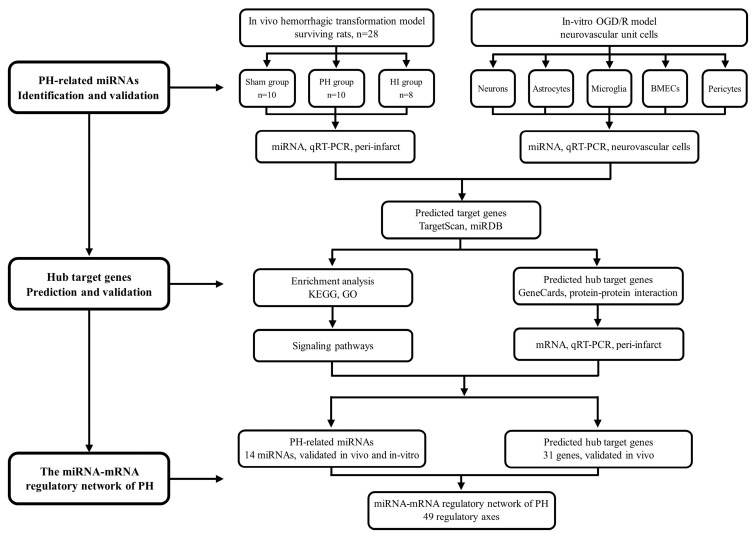
Program flowchart for this study. BMEC, brain microvascular endothelial cell; HI, hemorrhagic infarction; GO, Gene Ontology; KEGG, Kyoto Encyclopedia of Genes and Genomes; OGD/R, oxygen-glucose deprivation/reoxygenation; PH, parenchymal hematoma.

The purpose of this study is to bridge this gap by investigating the miRNA and mRNA regulatory networks associated with PH in a hyperglycemic ischemia–reperfusion rat model and an in vitro oxygen–glucose deprivation/reoxygenation (OGD/R) model of neurons, astrocytes, microglia, brain microvascular endothelial cells (BMECs), and pericytes. By elucidating these genetic pathways, we provide new evidence for cell-specific miRNA-mediated PH regulation and its associated pathways, including inflammation, oxidative stress, and BBB disruption, which are critical PH drivers. These findings advance our understanding of PH pathophysiology and may lay the groundwork for developing miRNA-based therapeutic strategies to improve outcomes in AIS patients undergoing EVT. The research strategy of this study is presented in Figure 1.

## 2. Materials and Methods

### 2.1. MCAO Model with Hemorrhagic Transformation After Mechanical Reperfusion

The Institutional Animal Care and Use Committee at our institution approved the animal experiment. We established a mechanical reperfusion-induced HT rat model under hyperglycemic conditions using adult male Sprague Dawley rats aged 8–10 weeks and weighing 250–280 g. The rats were anesthetized using 5% isoflurane in a chamber filled with a 70% nitrogen and 30% oxygen gas mix. Subsequently, the anesthesia was sustained with 2% isoflurane via a mask. Left middle cerebral artery occlusion (MCAO) was achieved using the intraluminal filament technique for 5 h, followed by 19 h of reperfusion, as previously described [23,24,25]. We prolonged the MCAO time to 5 h, resulting in severe cerebral ischemia, and we removed the filament to allow for instant reperfusion. This model mimicked cerebral blood flow changes and pathophysiological alterations upon reperfusion, similar to mechanical thrombectomy in humans [26]. An intraperitoneal injection of 50% dextrose (6 mL/kg) was given to the rats 15 min before MCAO to induce the condition of hyperglycemia with a glucose level higher than 16.7 mmol/L. Subsequently, 50% dextrose (1.5 mL/kg) was injected intraperitoneally at 1, 2, 3, and 4 h after MCAO to maintain the hyperglycemic state. The blood glucose level was monitored from the tail vein before the dextrose injection and at 0.5, 1, 2, and 4 h after the dextrose injection. The sham-operated hyperglycemic rats underwent the same surgical procedures without inserting the filament.

**Figure 2:**
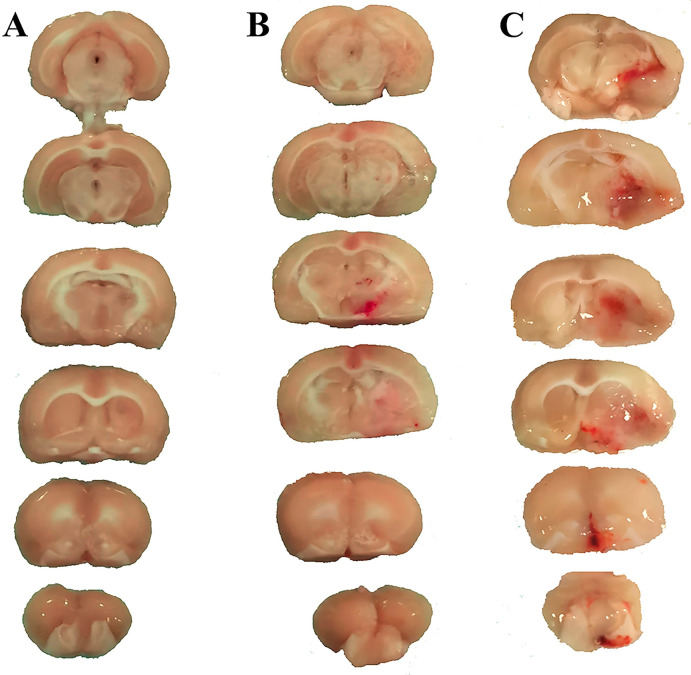
Macroscopic hemorrhagic transformation in the rat middle cerebral artery occlusion model was classified into no-hemorrhage (**A**), hemorrhagic infarction (**B**), and parenchymal hemorrhage (**C**).

Fifty-four rats with hyperglycemic states were used to create an animal model in this study. Two rats were excluded because of a subarachnoid hemorrhage after the surgery. Then, the 52 rats with hyperglycemic states were divided into an HT group (*n* = 42) and a sham-operated group (*n* = 10). The rats were sacrificed 19 h after the filament was removed (24 h after the onset of ischemia). This reperfusion-induced hemorrhage model had mortality within 19 h after reperfusion. Eighteen surviving rats in the transient MCAO group, comprising ten rats with PH and eight with hemorrhagic infarction, were further studied. Ten rats survived in the sham-operated group. Rat brains were harvested for measurements. HT was classified into hemorrhagic infarction and parenchymal hemorrhage, according to previous studies [23,24]. The coronal brain slices were used to assess macroscopic HT. Hemorrhagic infarction was defined as small or more confluent petechiae within the infarcted area or at the borders of the ischemic area. PH was shown as a large area of blood within the infarcted area (Figure 2).

### 2.2. Primary Cultures of Neurons, Astrocytes, Microglia, BMECs, and Pericytes

Sprague Dawley rat neurons, astrocytes, microglia, BMECs, and pericytes were prepared as in previous studies [27,28,29,30,31]. Cortical primary neurons were isolated from the cortex of fetal rats on days 16–18 of pregnancy. The cortex was digested with 0.25% trypsin (Gibco, Grand Island, NE, USA) at 37 °C for 15 min and incubated with DNaseI stock solution for 30 s. The cell suspension was collected using a 100 µM nylon cell filter and centrifuged at 800 rpm for 5 min. The cells were resuspended with high-sugar Dulbecco’s modified Eagle medium (DMEM) (Gibco) supplemented with 10% fetal bovine serum (FBS) (Gibco) and inoculated into a culture dish coated with 0.01% Poly-L-lysine (Sigma-Aldrich, Saint Louis, MO, USA). The cells were placed in a humidified incubator with 5% CO_2_ at 37 °C for 4 h and further cultured with a medium containing Neurobasal, 2% B27, 2% GlutaMAX, and 1% penicillin–streptomycin (Gibco). Half of the culture medium was replaced every three days. The primary neurons were cultured for eight days prior to OGD/R modeling.

Primary astrocytes were obtained from the cerebral cortex of neonatal rats aged 1–2 days. The cortical tissue was separated and digested using 0.25% trypsin at 37 °C for 20 min. A 70 µM nylon cell filter was used to collect the cell suspension, which was then centrifuged at 1000 rpm for 5 min. The cell pellet was resuspended in a complete medium containing DMEM-F12 (Gibco), 10% FBS, and 1% penicillin–streptomycin and seeded into 75 cm^2^ flasks. The cells were incubated in a humidified environment at 37 °C with 5% CO_2_, with the complete medium being refreshed every 2–3 days. The astrocytes were propagated to the third passage (14–16 days) before OGD/R treatment.

Primary microglia were isolated from the cortex of newborn rats on days 1–2. Like the astrocytes above, the cortex was digested, and the resulting cell suspension was collected using a filter. Then, the cell suspension was centrifuged at 1500 rpm for 5 min. The cells were resuspended and placed into 0.01% Poly-L-lysine pre-coated 75 cm^2^ flasks. The cells were cultured in a humidified incubator with 5% CO_2_ at 37 °C for two weeks, with the complete medium being replaced every three days. Subsequently, the loosely attached microglia were isolated by shaking the flasks for 4 h at 200 rpm on a rotary shaker at 37 °C. The cell suspension was then collected and centrifuged at 2000 rpm for 5 min. The cells were resuspended and cultured with the complete medium. The primary microglia were cultured for two days before OGD/R treatment, resulting in a total culture period of 16 days.

Primary BMECs were isolated from the cortex of rats on weeks 1–2. The cortex was sequentially digested with 0.1% type IV collagenase with 30 U/mL DNase I (Gibco) at 37 °C for 1.5 h and 0.1% collagenase/dispase enzyme containing 20 U/mL DNase I (Sigma-Aldrich) for 1 h. The cell suspension was centrifuged at 1000 rpm for 8 min. The cells were suspended with DMEM, spread on 50% Percoll (Sigma-Aldrich) with a continuous gradient formed via centrifugation, and centrifuged at 25,000× *g* for 1 h. The purified microvascular segments were collected, rinsed, and centrifuged at 1000 rpm for 5 min. The cells were resuspended with the endothelial cell medium (ECM) (ScienCell, San Diego, CA, USA), placed into 25 cm^2^ flasks coated with type I collagen (Corning, Corning, NY, USA), and cultured in a humidified incubator with 5% CO_2_ at 37 °C for 24 h. Then, the cells were cultured with the ECM containing 4 µg/mL puromycin (Sigma-Aldrich) for 48 h and replaced with the ECM without puromycin. After that, the ECM was changed every three days. The BMECs were expanded to the third passage (15–16 days) before OGD/R treatment.

Primary pericytes were isolated from the cortex of rats on weeks 2–3. The cortex was isolated, minced, and homogenized in pre-cooled PBS, and then the homogenate was centrifuged at 500 rpm for 5 min at 4 °C. The precipitation was suspended in 20% Percoll and centrifuged at 500 rpm for 20 min at 4 °C. Next, the sediment was collected and digested with 0.1% collagenase II and 1000 U/mL DNase I at 37 °C for 1 h. After centrifugation at 500 rpm for 5 min at 4 °C, the precipitation was harvested, resuspended in 20% Percoll, and centrifuged at 500 rpm for 15 min at 4 °C. Subsequently, the middle layer was collected and centrifuged at 500 rpm for 5 min at 4 °C. The sediment was resuspended with the ECM for pericyte (ScienCell). The cells were cultured in a humidified incubator with 5% CO_2_ at 37 °C, with the ECM for pericyte being changed every three days. The pericytes were cultured to the third passage (12–13 days) and subsequently utilized for the OGD/R experiment.

The purity of the primary neurons, astrocytes, microglia, BMECs, and pericytes was determined using immunofluorescence before subsequent experiments, and the purity of all types of cells was greater than 90%.

### 2.3. OGD/R Model

The rat neuron, astrocyte, microglia, BMEC, and pericyte cultures were separately exposed to OGD for a fixed time [27,28,29,30,31]. The in vitro OGD/R model mimics ischemic conditions by depriving cells of oxygen and glucose, simulating the metabolic environment of ischemia observed in vivo. These complementary approaches allowed us to investigate the shared and cell-specific mechanisms underlying PH after mechanical reperfusion in acute ischemia. The cell cultures were placed into a hypoxic chamber (Stemcell, Vancouver, BC, Canada) at 37 °C with a gas mixture of 95% N_2_ and 5% CO_2_ and cultured in a glucose-free medium. The neurons were maintained in the hypoxic chamber for 1 h [27]. The astrocytes were maintained in the hypoxic chamber for 6 h [28]. The BMECs, pericytes, and microglia were separately maintained in the hypoxic chamber for 2 h [29,30,31]. Then, the cells were transferred to a cell incubator under normoxic conditions in a complete medium with normal glucose for reoxygenation and glucose restoration mimicking reperfusion conditions. The neurons were collected at the time of reoxygenation at 0, 6, 12, 24, 48, and 72 h. The astrocytes, microglia, BMECs, and pericytes were collected during reoxygenation at 0, 3, 6, 12, and 24 h. The cells in the control group did not receive OGD. The differential expression of PH-related miRNAs in the OGD/R model at the above reoxygenation times was further assessed.

### 2.4. RNA Extraction and Quantitative RT-PCR

Twenty-four hours after MCAO, the brains of the rats were promptly extracted, and the peri-infarct and infarction core tissues from the ischemic hemisphere were isolated as in previous studies [32,33]. The midline between the ischemic and non-ischemic hemispheres was identified. A longitudinal cut from top to bottom along the sagittal plane at a distance of 2 mm from the midline through each hemisphere was made. Then, a transverse oblique cut at approximately the “2 o’clock” position was performed to separate the brain tissue from the infarction core (the striatum and upper cerebral cortex) and the peri-infarct area (the adjacent cerebral cortex). Total RNA was extracted from the ischemic hemisphere’s peri-infarct and infarction core tissues using Trizol reagent (Invitrogen, Carlsbad, CA, USA). Total RNA was extracted from the rat neurons, astrocytes, microglia, BMECs, and pericytes after OGD/R. Good RNA quality was considered an OD260/OD280 ratio between 1.8 and 2.0.

Twenty-nine significantly differentially expressed miRNAs in the ischemic hemispheres from the rat reperfusion-induced HT model examined using a miRNA array was preliminarily shown in our previous study [23]. Twenty-five differentially expressed miRNAs identified in the miRNA array, all expressed in rats, humans, and mice, were assessed using quantitative RT-PCR in the current study. The other four miRNAs (miR-344a-3p, miR-434-3p, miR-3557-5p, and miR-3596b) only expressed in rats (not expressed in humans or mice) were excluded. The twenty-five miRNAs were miR-1-3p, miR-25-5p, miR-27b-3p, miR-29a-5p, miR-29c-3p, miR-30a-3p, miR-126a-5p, miR-132-3p, miR-136-3p, miR-138-5p, miR-139-5p, miR-142-3p, miR-153-5p, miR-195-3p, miR-218a-5p, miR-219a-2-3p, miR-320-5p, miR-337-3p, miR-369-5p, miR-376a-5p, miR-376b-5p, miR-381-3p, miR-383-5p, miR-487b-5p, and miR-582-3p. The differentially expressed miRNAs among the sham-operated (*n* = 10), PH (*n* = 10), and hemorrhagic infarction groups (*n* = 6) were assessed. Then, significantly differentially expressed PH-related miRNAs confirmed in both the peri-infarct and infarction core tissues in the MCAO model were further assessed in the OGD/R model of neurons, astrocytes, microglia, BMECs, and pericytes.

The expression levels of mature miRNAs and mRNAs of predicted target genes from the later bioinformatics analysis of the peri-infarct and infarction core tissues and cells after OGD/R were assessed using the TaqMan stem-loop and the SYBR green real-time RT-PCR method. We performed quantitative RT-PCR using the PCR Master Mix on the Applied Biosystem PCR System (Thermo Fisher Scientific, Waltham, MA, USA). MiRNA and mRNA expression levels were normalized using U6 and β-actin as internal controls and were calculated using the 2^−ΔΔCT^ method. The primer sequences for the miRNAs and predicted target mRNAs are shown in Tables S1 and S2.

### 2.5. Gene Ontology (GO) and Kyoto Encyclopedia of Genes and Genomes (KEGG) Pathway Analyses

The significantly differentially expressed PH-related miRNAs confirmed in the peri-infarct of the ischemic hemisphere in the rat MCAO model were selected for a bioinformatics analysis and target gene prediction. We searched the predicted target genes of the differentially expressed miRNAs using the TargetScan and miRDB databases. KEGG pathway and GO functional enrichment analyses were carried out to reveal the biological function of the predicted target genes using the cluster Profiler R package (https://yulab-smu.top/biomedical-knowledge-mining-book/; version 4.11.0; accessed on 5 January 2024) [34]. The biological process, cellular component, and molecular function of the predicted target genes were revealed in the GO enrichment analysis. KEGG and GO terms with an adjusted *p* value of less than 0.05 were considered significantly enriched.

### 2.6. Construction of miRNA-mRNA Regulatory Network of PH

We obtained the immune-, inflammation-, oxidative stress-, and apoptosis-related genes from the GeneCards database (http://www.genecards.org; version 5.18; accessed on 24 November 2023). The Search Tool for the Retrieval of Interacting Genes (STRING) (https://cn.string-db.org/cgi/input; version 11.5; accessed on 26 November 2023) was applied to identify the protein–protein interaction network of the predicted target genes related to the mechanisms of immunity, inflammation, oxidative stress, and apoptosis. The top twenty hub genes associated with immune, inflammatory, oxidative stress, and apoptosis mechanisms were identified using the maximal clique centrality algorithm in the CytoHubba, a plugin in Cytoscape software (version 3.9.1). Then, the predicted miRNA-mRNA network containing hub genes was established using the online tool Cytoscape (https://cytoscape.org; version 3.9.1; accessed on 26 November 2023). Finally, we obtained the miRNA-mRNA regulatory network of PH after the expression of the predicted hub genes was confirmed via RT-PCR in the rat reperfusion-induced PH model.

### 2.7. Statistical Analysis

We performed statistical analyses using SPSS 22.0 software. The variables were analyzed using the Student’s *t*-test. A one-way ANOVA was performed for multiple comparisons using Dunnett’s test. A *p* value of less than 0.05 was considered significant.

## 3. Results

### 3.1. MiRNAs in Rat Ischemic Brain with PH After Mechanical Reperfusion

**Figure 3:**
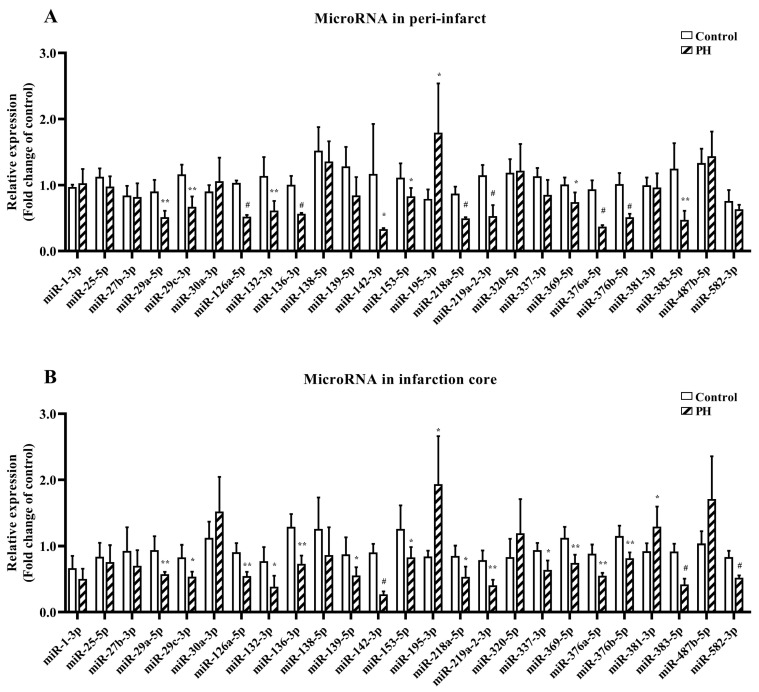
Twenty-five microRNAs shown from our previous microRNA array study were confirmed in the peri-infarct (**A**) and infarction core (**B**) by quantitative RT-PCR. Fourteen microRNAs were significantly differentially expressed in the rats with parenchymal hemorrhage (*n* = 5) compared with the sham-operated rats (*n* = 5), including 13 down-regulated microRNAs and one up-regulated microRNA. MiR, microRNA; PH, parenchymal hemorrhage. Results were expressed as Mean ± SD. Statistical comparisons were done with the Student *t*-test. * *p* < 0.05; ** *p* < 0.01; ^#^ *p* < 0.001.

HT was observed in all 42 hyperglycemic rats receiving 5 h MCAO and mechanical reperfusion after 19 h. The percentage of PH was 43% in the transient MCAO group. This reperfusion-induced hemorrhage model had a 57% mortality rate, mainly within 12 h after reperfusion. Eighteen rats in the transient MCAO group, comprising ten rats with PH and eight rats with hemorrhagic infarction, survived. Ten rats in the sham-operated group also survived. Twenty-five differentially expressed miRNAs identified in our previous miRNA array study were further confirmed via quantitative RT-PCR. Finally, 14 miRNAs were significantly differentially expressed in both the peri-infarct and infarction core in the rats with PH compared with the sham-operated rats, comprising 13 down-regulated miRNAs and one up-regulated miRNA (miRNA-195-3p). These 13 down-regulated miRNAs associated with PH were miRNA-126a-5p, miRNA-132-3p, miRNA-136-3p, miRNA-142-3p, miRNA-153-5p, miRNA-218a-5p, miRNA-219a-2-3p, miRNA-29a-5p, miRNA-29c-3p, miRNA-369-5p, miRNA-376a-5p, miRNA-376b-5p, and miRNA-383-5p (Figure 3; Table 1). Of the 14 PH-related miRNAs, 10 did not show a significant difference in expression between the peri-infarct or infarction core in the rats with hemorrhagic infarction and those in the sham-operated group. These ten miRNAs were miRNA-126a-5p, miRNA-132-3p, miRNA-136-3p, miRNA-142-3p, miRNA-153-5p, miRNA-218a-5p, miRNA-29a-5p, miRNA-29c-3p, miRNA-369-5p, and miRNA-376b-5p. The remaining PH-related miRNAs showed a trend toward greater differential expression in the PH group than those in the rats with hemorrhagic infarction (Figure S1).

### 3.2. Differential Expression of PH-Related miRNAs in the OGD/R Model

These 14 significantly differentially expressed miRNAs associated with PH in the rat MCAO model were further assessed in the OGD/R model with neurons, astrocytes, microglia, BMECs, and pericytes (Table 1 and Figures S2–S6).

We identified 11 miRNAs that had significantly different abundance levels at 24 h in the neurons treated with OGD/R compared with the non-OGD/R control. Eight down-regulated miRNAs were consistent with the results of the animal model: miRNA-132-3p, miRNA-136-3p, miRNA-153-5p, miRNA-218a-5p, miRNA-369-5p, miRNA-376a-5p, miRNA-376b-5p, and miRNA-383-5p (Figure S2).

We identified ten miRNAs that had significantly different abundance levels at 24 h in the OGD/R-treated astrocytes compared with the non-OGD/R control, all of which were decreased and consistent with the animal model results. These 10 down-regulated miRNAs were miRNA-126a-5p, miRNA-132-3p, miRNA-142-3p, miRNA-153-5p, miRNA-218a-5p, miRNA-219a-2-3p, miRNA-29a-5p, miRNA-29c-3p, miRNA-369-5p, and miRNA-383-5p (Figure S3).

Six miRNAs had significantly different abundance levels at 24 h in the OGD/R-treated microglia compared with the non-OGD/R control. Three down-regulated miRNAs were consistent with the results of the animal model: miRNA-132-3p, miRNA-136-3p, and miRNA-218a-5p (Figure S4).

We identified seven miRNAs with significantly different abundance levels at 24 h in the OGD/R-treated BMECs compared with the non-OGD/R control. Three down-regulated miRNAs (miRNA-126a-5p, miRNA-142-3p, and miRNA-29c-3p) and one up-regulated miRNA (miRNA-195-3p) were consistent with the results of the MCAO model (Figure S5).

Seven miRNAs had significantly different abundance levels at 24 h in the OGD/R-treated pericytes compared with the non-OGD/R control. Two down-regulated miRNAs (miRNA-132-3p and miRNA-218a-5p) and one up-regulated miRNA (miRNA-195-3p) were consistent with the results of the MCAO model (Figure S6).

**Figure 4:**
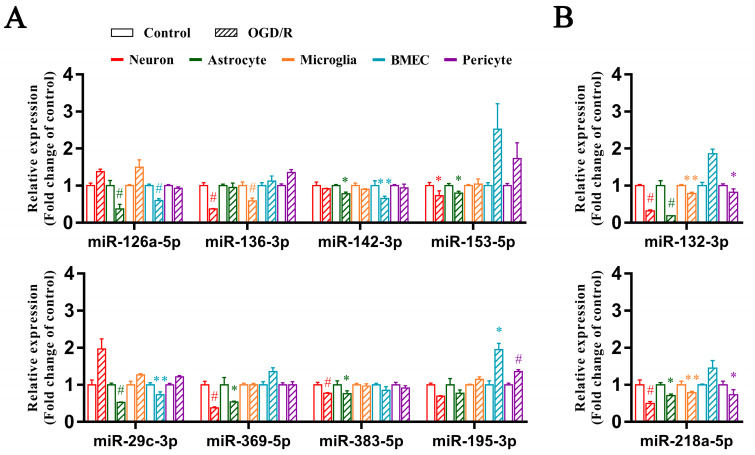
Ten PH-associated microRNAs were significantly differentially expressed in at least two of five models of neurons, astrocytes, microglia, brain microvascular endothelial cells (BMECs), and pericytes after oxygen-glucose deprivation/reoxygenation (OGD/R) at 24 h. (**A**) eight microRNAs (miRNA-126a-5p, miRNA-136-3p, miRNA-142-3p, miRNA-153-5p, miRNA-195-3p, miRNA-29c-3p, miRNA-369-5p, miRNA-383-5p) were significantly differentially expressed in two cell models of OGD/R. (**B**) two microRNAs (miRNA-132-3p, miRNA-218a-5p) were significantly altered in four cell models of OGD/R. Results were expressed as Mean ± SD from three independent experiments. Statistical comparisons were performed with one-way ANOVA for the groups of different reoxygenation times. * *p* < 0.05; ** *p* < 0.01; ^#^ *p* < 0.001.

Ten miRNAs were significantly differentially expressed in at least two of the five neuron, astrocyte, microglia, BMEC, and pericyte models after OGD/R at 24 h, consistent with the results of the animal model (Figure 4). MiRNA-126a-5p, miRNA-142-3p, and miRNA-29c-3p significantly decreased in the astrocyte and BMEC model after OGD/R at 24 h. MiRNA-153-5p, miRNA-369-5p, and miRNA-383-5p significantly decreased in the neuron and astrocyte model after OGD/R at 24 h. MiRNA-136-3p significantly decreased in the neuron and microglia model after OGD/R at 24 h. MiRNA-195-3p significantly increased in the BMEC and pericyte model after OGD/R at 24 h. Both miRNA-132-3p and miRNA-218a-5p significantly decreased in the model of neurons, astrocytes, microglia, and pericytes after OGD/R at 24 h.

### 3.3. KEGG Pathway and GO Analysis of Differentially Expressed miRNAs Associated with PH

**Figure 5:**
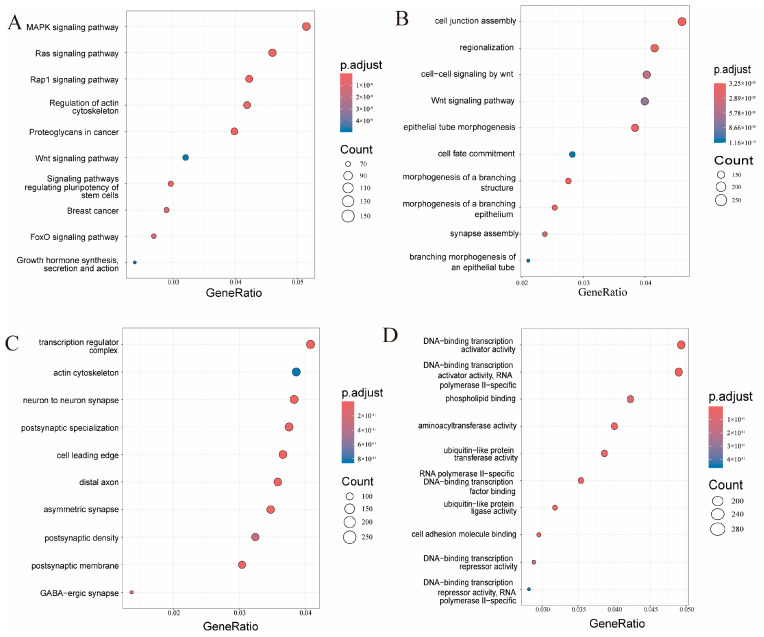
Kyoto Encyclopedia of Genes and Genomes (KEGG) pathway and Gene Ontology (GO) functional enrichment analysis of the predicted target genes from differentially expressed microRNAs. (**A**) KEGG. (**B**) GO-biological processes. (**C**) GO-cell components. (**D**) GO-molecular functions.

To identify the potential biological function of these 14 differentially expressed miRNAs, we found 9992 target genes for these miRNAs predicted using TargetScan and miRDB databases. Then, KEGG and GO enrichment analyses were performed. The top ten enriched terms of the KEGG pathways, biological process, cellular component, and molecular function of GO were archived (Figure 5). The top ten enriched pathways were as follows: MAPK signaling pathway, Ras signaling pathway, Rap1 signaling pathway, regulation of actin cytoskeleton, proteoglycans in cancer, Wnt signaling pathway, signaling pathways regulating pluripotency of stem cells, breast cancer, FoxO signaling pathway, growth hormone synthesis, secretion and action.

### 3.4. Differential Expression of Predicted Target Genes in the Rat PH Model

**Figure 6:**
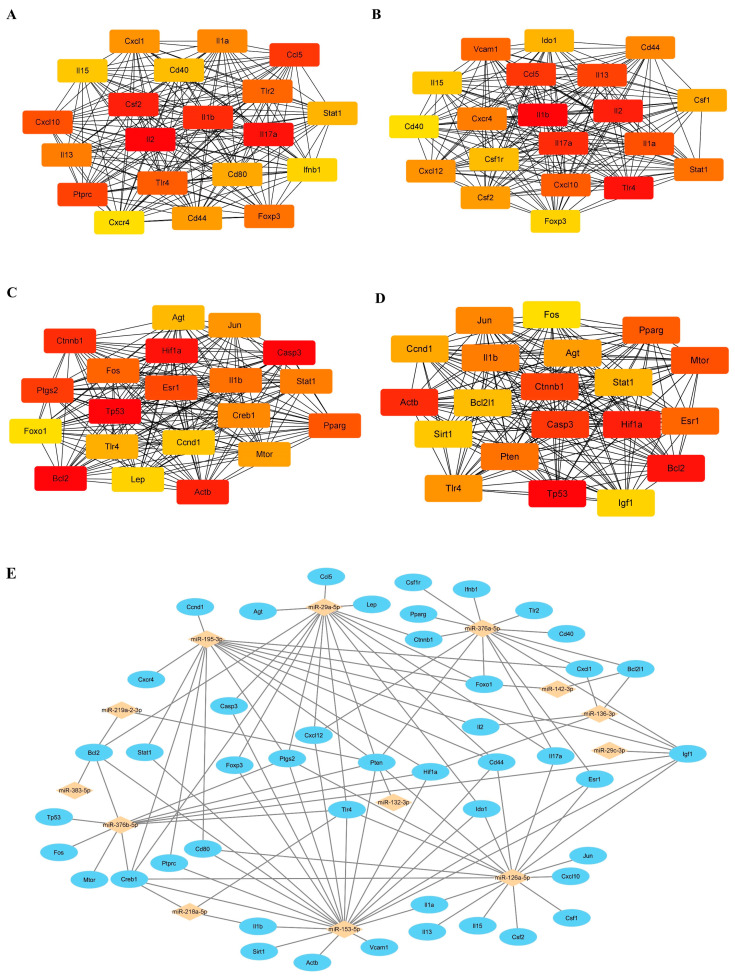
The predicted microRNA-mRNA regulatory network of PH. Relationship network diagram of the top twenty hub genes related to immune (**A**), inflammation (**B**), oxidative stress (**C**), and apoptosis (**D**) from the protein-protein interaction network. (**E**) The reconstructed 107 microRNA-mRNA regulatory axes with 46 predicted hub genes.

The immune-, inflammation-, oxidative stress-, and apoptosis-related genes were obtained from the GeneCards database. There were 633, 507, 565, and 811 predicted target genes related to immunity, inflammation, oxidative stress, and apoptosis, respectively. Using the STRING online tool, we identified the protein–protein interaction network of these predicted target genes related to immunity, inflammation, oxidative stress, and apoptosis. Then, the top twenty hub genes related to immunity, inflammation, oxidative stress, and apoptosis were calculated using the CytoHubba plugin, as shown in Figure 6A–D, respectively. A total of 46 key predicted target genes for these 14 PH-related miRNAs were identified, as 28 genes were involved in at least two mechanisms of immunity, inflammation, oxidative stress, and apoptosis. Then, we predicted the miRNA-mRNA network of PH, including 107 miRNA-mRNA regulatory axes containing 46 hub genes, using Cytoscape software (version 3.9.1) (Figure 6E).

**Figure 7:**
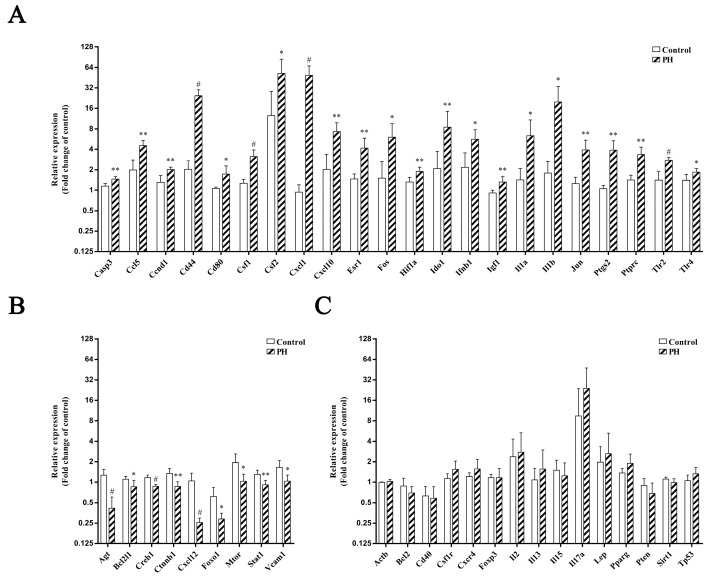
Forty-six predicted hub genes from differentially expressed microRNAs were assessed using quantitative RT-PCR in the peri-infarct. Thirty-one genes were significantly differentially expressed in peri-infarct tissue in rats with parenchymal hemorrhage (PH) (*n* = 5) compared with sham-operated rats (*n* = 5), including 22 up-regulated genes (**A**) and nine down-regulated genes (**B**). Fifteen genes were not significantly differentially expressed in peri-infarct tissue between the two groups (**C**). Results were expressed as Mean ± SD. Statistical comparisons were performed with the Student *t*-test. * *p* < 0.05; ** *p* < 0.01; ^#^ *p* < 0.001.

These 46 predicted hub genes were further assessed via RT-PCR in the animal model. Thirty-one genes were significantly differentially expressed in the peri-infarct tissue in the rats with PH compared with the sham-operated rats (Figure 7). Twenty-two up-regulated genes associated with PH were confirmed, including caspase 3 (Casp3), chemokine C-C motif chemokine ligand 5 (Ccl5), cyclin D1 (Ccnd1), CD44 molecule (Cd44), Cd80, colony stimulating factor (Csf)1, Csf2, C-X-C motif chemokine ligand (Cxcl) 1, Cxcl10, estrogen receptor (Esr) 1, Fos proto-oncogene, AP-1 transcription factor subunit (Fos), hypoxia inducible factor 1 alpha subunit (Hif1a), indoleamine 2,3-dioxygenase 1 (Ido1), interferon beta 1 (Ifnb1), insulin-like growth factor 1 (Igf1), interleukin 1 alpha (Il1a), interleukin 1 beta (Il1b), jun proto-oncogene, AP-1 transcription factor subunit (Jun), prostaglandin-endoperoxide synthase 2 (Ptgs2), protein tyrosine phosphatase receptor type C (Ptprc), toll-like receptor 2 (Tlr2), and toll-like receptor 4 (Tlr4). Nine down-regulated genes associated with PH were angiotensinogen (Agt), BCL2-like 1 (Bcl2l1), cAMP responsive element binding protein 1 (Creb1), catenin beta 1 (Ctnnb1), Cxcl12, forkhead box O1 (Foxo1), mechanistic target of rapamycin kinase (Mtor), signal transducer and activator of transcription 1 (Stat1), and vascular cell adhesion molecule 1 (Vcam1). Finally, we confirmed twenty-five, twenty-two, twenty, and twenty-five PH-associated genes involved in the mechanisms of immunity, inflammation, oxidative stress, and apoptosis, respectively. Eight up-regulated genes (Ccl5, Fos, Hif1a, Il1a, Il1b, Jun, Ptgs2, and Tlr4) and four down-regulated genes (Creb1, Ctnnb1, Mtor, and Stat1) were involved in all mechanisms of immunity, inflammation, oxidative stress, and apoptosis.

### *3.5. The* miRNA-mRNA Regulatory Network of PH

**Figure 8:**
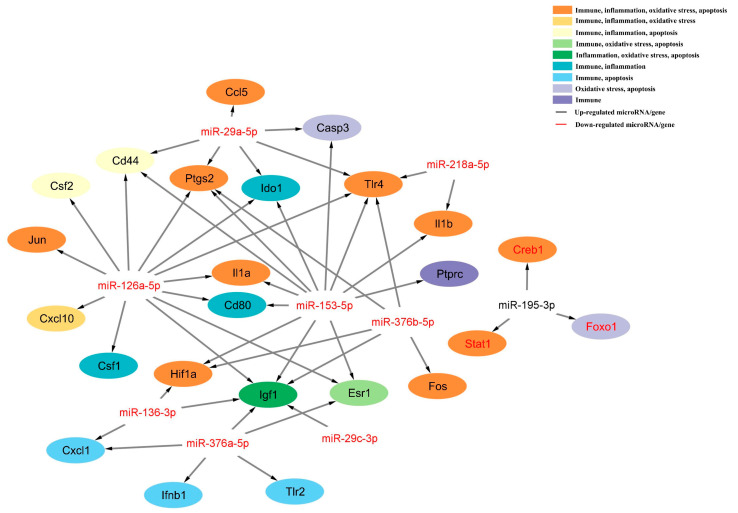
The microRNA-mRNA regulatory axes of parenchymal hematoma based on the prediction tool and PCR examination, including nine microRNAs (eight down-regulated microRNAs (in red) and one up-regulated microRNA (in black)) and their 24 essential target genes (three down-regulated genes and 21 up-regulated genes) with 49 microRNA-mRNA regulatory axes. This network was associated with the mechanism of immune, inflammation, oxidative stress, and apoptosis.

Finally, we obtained the miRNA-mRNA network of PH based on the prediction tool and PCR examination, including nine miRNAs and their 24 essential target genes with 49 miRNA-mRNA regulatory axes (Figure 8). The twenty-four essential target genes were Casp3, Ccl5, Cd44, Cd80, Creb1, Csf1, Csf2, Cxcl1, Cxcl10, Esr1, Fos, Foxo1, Hif1a, Ido1, Ifnb1, Igf1, Il1a, Il1b, Jun, Ptgs2, Ptprc, Stat1, Tlr2, and Tlr4. Nine of the above top ten enriched pathways were involved, including MAPK signaling pathway, Ras signaling pathway, Rap1 signaling pathway, proteoglycans in cancer, Wnt signaling pathway, signaling pathways regulating pluripotency of stem cells, breast cancer, FoxO signaling pathway, growth hormone synthesis, secretion and action. Forty-nine miRNA-mRNA regulatory axes of PH were associated with the mechanisms of immunity, inflammation, oxidative stress, and apoptosis. These nine microRNAs and their predicted target genes, along with the involved mechanisms and signaling pathways, are presented in supplementary Table S3 and Figure S7.

## 4. Discussion

This study found 13 down- and one up-regulated miRNAs in both the peri-infarct and infarction core with PH from the hyperglycemic rat MCAO model after mechanical reperfusion. Among these 14 PH-related miRNAs, 10 were significantly differentially expressed in at least two of the five neuron, astrocyte, microglia, BMEC, and pericyte models after OGD/R. The MAPK, Ras, Rap1, and Wnt signaling pathways were the enriched pathways of these PH-related miRNAs. MiRNAs contribute to cerebral ischemic injury by regulating oxidative stress, inflammation, apoptosis, and BBB permeability [22,35,36,37]. In this study, we found 31 significantly differentially expressed target genes related to immunity, inflammation, oxidative stress, and apoptosis, which were predicted from these PH-associated miRNAs. Forty-nine miRNA-mRNA regulatory axes of PH were revealed.

Among these 14 PH-related miRNAs, eight have been reported as potential regulators of cerebral ischemia or reperfusion injury in previous studies [19,22,35,36,37]. Among the 14 PH-related miRNAs identified, miRNA-126a-5p emerged as a central regulator of BBB integrity, highlighting its therapeutic potential in mitigating ischemic stroke complications. Our study found that miRNA-126-5p decreased in the peri-infarct of the rat MCAO model with PH and BMECs after OGD/R at 24 h, suggesting its pivotal role in maintaining endothelial homeostasis. Previous studies have shown that miRNA-126a-5p modulates endothelial cell function by targeting pathways critical for angiogenesis and vascular integrity, such as the MAPK and PI3K/Akt signaling pathways [38]. Its down-regulation has been associated with increased vascular permeability and BBB disruption, which are hallmark features of PH. In our study, the reduced expression of miRNA-126a-5p correlated with the up-regulation of inflammatory and apoptotic genes, further supporting its role as a protector of BBB integrity under ischemic–reperfusion conditions.

Our study provides new evidence for the association of miRNAs with PH in AIS after mechanical reperfusion therapy. Most differentially expressed miRNAs were not reported to be involved in PH after reperfusion [20,21,39,40,41,42,43,44]. The overexpression of miRNA-21-5p, miRNA-206, and miRNA-3123 was associated with HT in patients with cardioembolic stroke. These three miRNAs, related to matrix metalloproteinase-9, may serve as prognostic blood markers for HT in cardioembolic stroke [20,39]. A recent study revealed the expression levels of non-coding RNA in peripheral blood mononuclear cells associated with HT in six patients with AIS receiving EVT. There were 47 significantly differentially expressed miRNAs in the peripheral blood of patients with HT compared with those without HT. MiRNA-383-5p was one of the top ten down-regulated miRNAs related to HT, similar to our study [21]. In a transient MCAO model of wild-type mice, miRNA-155 increased in the peri-infarct regions, which regulated BBB permeability via direct interactions with several mRNAs. The genetic deletion of miRNA-155 significantly reduced the hemorrhagic burden after cerebral ischemic reperfusion [40]. In addition, we found 10 PH-related miRNAs significantly differentially expressed in at least two of the five neuron, astrocyte, microglia, BMEC, and pericyte models after OGD/R. Six PH-associated miRNAs (miRNA-126a-5p, miRNA-132-3p, miRNA-142-3p, miRNA-195-3p, miRNA-29c-3p, and miRNA-218a-5p) had significant differential expression in the model of BMECs or pericytes after OGD/R.

Recent studies have suggested that the pathophysiology of cellular excitotoxicity, cell death signaling, oxidative stress and mitochondrial dysfunction, neuroinflammation, and BBB disruption play a pivotal role in cerebral ischemia. Immune cells in the brain and peripheral blood participate in the acute and subacute pathogenesis of ischemic stroke. Therapies targeting the signaling pathways involved in these molecular mechanisms provide potential strategies for treating ischemic stroke and alleviating ischemic brain injury [45,46,47]. Modulating inflammatory and immune responses may be a potential crucial intervention to alleviate BBB disruption and HT in ischemic stroke [48]. In our study, we identified several key pathways and hub genes that play significant roles in the pathophysiology of PH following endovascular mechanical reperfusion in AIS. Specifically, the MAPK, Ras, and Wnt signaling pathways were among the most enriched, indicating their critical involvement in mediating cellular responses to ischemic injury and subsequent reperfusion. The MAPK signaling pathway, for instance, is well known for its role in regulating cell growth, differentiation, and response to stress, and its activation has been implicated in promoting inflammatory responses and apoptosis following ischemic stroke [35,45]. Similarly, the Ras signaling pathway is essential for cell proliferation and survival, and its dysregulation can lead to increased oxidative stress and neuronal damage [36]. The Wnt signaling pathway, however, is involved in maintaining BBB integrity and neuronal survival, with disruptions in this pathway contributing to increased permeability and HT [38]. By highlighting these pathways, we provide a mechanistic understanding of how molecular signals contribute to the pathological processes observed in PH, emphasizing the need for targeted therapeutic strategies to mitigate these effects.

Our study confirmed that 31 predicted hub target genes related to oxidative stress, apoptosis, immunity, and inflammation may be associated with PH in the MCAO model after mechanical reperfusion. Twenty genes were involved in the mechanisms of immunity and/or inflammation. Twelve genes were associated with all mechanisms of oxidative stress, apoptosis, immunity, and inflammation. Twelve genes have been reported as potential regulators of HT in ischemic stroke in previous studies: Casp3, Csf2, Ctnnb1, Cxcl12, Hif1a, Il1b, Mtor, Ptgs2, Ptprc, Tlr2, Tlr4, and Vcam1 [41,42,49,50,51,52,53,54,55,56,57,58]. The other 19 genes may be potential therapy targets for improving PH in AIS after mechanical reperfusion treatment. Notably, genes such as Casp3, Il1b, and Tlr4 were up-regulated, implicating them in the inflammatory and apoptotic pathways that exacerbate neuronal injury and BBB disruption [46,57]. Casp3, a key executor of apoptosis, has been shown to mediate cell death in ischemic stroke, while Il1b and Tlr4 are pivotal in initiating and propagating inflammatory responses [47,49]. The up-regulation of these genes suggests their role in promoting secondary brain injury following ischemic stroke and reperfusion.

Valuable miRNAs can regulate several target genes involved in multiple PH-related mechanisms. Simultaneously differentially expressed PH-associated miRNAs in neurons, astrocytes, microglia, BMECs, and pericytes under ischemic–reperfusion conditions may serve as therapeutic targets to alleviate ischemic brain injury. Recent studies showed that miRNA-218-5p expression increased in astrocytes during OGD/R. The inhibition of miRNA-218-5p reduced the inflammation, oxidative stress, and apoptosis damage induced by ischemic reperfusion injury [59,60]. In our study, the differential expression of miRNA-218a-5p and its two key predicted target genes of Il1b and Tlr4 were found in the peri-infarct of the rat PH model and the in vitro OGD/R model of neurons, astrocytes, microglia, and pericytes (Figure S8). Both Il1b and Tlr4 were involved in all immune, inflammatory, oxidative stress, and apoptotic mechanisms. MiRNA-195-5p was significantly down-regulated during cerebral ischemic reperfusion injury in the OGD/R model of BMECs and the MCAO model. An increased expression of miRNA-195-5p alleviated cerebral ischemic reperfusion injury [61]. In our study, the expression of miRNA-195-3p was up-regulated, and that of its three key predicted target genes of Creb1, Foxo1, and Stat1 decreased in the peri-infarct of the rat PH model. The differential expression of miRNA-195-3p and these predicted target genes was found in the BMECs and pericytes after OGD/R (Figure S9). Creb1 is involved in the mechanism of intracerebral hemorrhage by regulating inflammation and BBB disruption [62]. Foxo1 may be involved in OGD/R-induced injury in BMECs and may play a role in early brain injury after subarachnoid hemorrhage [63,64]. By elucidating these interactions, our study provides insights into the molecular underpinnings of PH, offering potential biomarkers for early detection and targets for therapeutic intervention.

The differential expression of miRNAs observed in this study reflects their distinct roles in regulating the molecular pathways underlying PH after ischemia–reperfusion injury. Among the 14 identified PH-related miRNAs, 13 were down-regulated, and one was up-regulated, suggesting that ischemic stress and reperfusion-induced injury disrupt the tightly regulated balance of miRNA-mediated gene expression. The down-regulation of protective miRNAs, such as miRNA-126a-5p and miRNA-132-3p, likely reflects a loss of their regulatory functions in maintaining vascular stability, controlling inflammation, and reducing oxidative stress. Their reduced expression in PH may exacerbate BBB disruption, allowing inflammatory cells and blood components to infiltrate the brain parenchyma, contributing to hematoma growth. In addition, the down-regulation of miRNAs may amplify neuronal and vascular cell death, thereby worsening ischemic injury and promoting hemorrhagic transformation. In contrast, the up-regulation of miRNA-195-3p suggests a compensatory response to ischemic stress. miRNA-195-3p has been implicated in regulating apoptotic pathways, where increased expression may reflect an attempt to counteract cell death in the early stages of reperfusion injury. However, the excessive or prolonged up-regulation of miRNA-195-3p may dysregulate its target pathways, including Creb1 and Foxo1, which are critical for BBB repair and cellular resilience. This dysregulation could paradoxically exacerbate secondary injury. Understanding why specific miRNAs are down- or up-regulated provides critical insights into the pathophysiology of PH. It also highlights therapeutic opportunities to restore balance: reintroducing down-regulated protective miRNAs (e.g., miRNA-126a-5p) or attenuating the effects of up-regulated miRNAs (e.g., miRNA-195-3p) could mitigate BBB damage and reduce PH risk.

Our study suggests the therapeutic potential of miRNA modulation in reducing PH after mechanical reperfusion in acute ischemic stroke. Using miRNA mimics can restore the expression of protective miRNAs, which will enhance vascular stability and reduce inflammatory responses. In contrast, miRNA inhibitors can suppress deleterious miRNAs to mitigate further damage. Using nanoparticle systems, exosome-based carriers, and viral vectors may offer a promising solution for effectively delivering miRNA-based therapies.

This study has some limitations. We assessed the expression of PH-related miRNAs in neurons, astrocytes, microglia, BMECs, and pericytes after OGD/R. However, each miRNA may be involved in the cross-talk process in the interaction of these cells during cerebral ischemia–reperfusion. Neurovascular unit cells with high glucose exposure in the OGD/R model can resemble the pathophysiological status of hemorrhagic transformation in the hyperglycemic ischemia–reperfusion animal model [65,66,67]. In addition, this study did not evaluate the direct interactions of these PH-related miRNAs with their target genes using dual luciferase reporter technology. The exact intervening effect of these differentially expressed miRNAs on PH through regulating their target genes requires further study through animal and OGD/R models with normal glucose and high glucose exposure. Finally, the lack of human validation data represents a limitation.

The findings of our study have significant translational potential for acute ischemic stroke in clinical settings. The identified miRNAs are potential biomarkers for the early detection of PH after mechanical reperfusion and targets for therapeutic interventions for PH. The altered expression of these potential miRNAs in blood or cerebrospinal fluid could facilitate non-invasive risk stratification and enable therapeutic interventions. Future studies will be essential to validate these findings in larger animal models and conduct pilot trials on human acute stroke patients. Such studies will be necessary to assess the utility of miRNA-based biomarkers and the therapeutic potential of miRNAs, paving the way for personalized interventions in AIS.

## 5. Conclusions

Fourteen miRNAs were found to be associated with PH after mechanical reperfusion in rat MCAO and in vitro OGD/R models. Simultaneously differentially expressed miRNAs and their associated genes in neurons, astrocytes, microglia, BMECs, and pericytes may serve as valuable targets for PH after endovascular mechanical reperfusion in AIS. The vital role of reperfusion-inducible miRNAs and their related target genes via the signaling pathway in PH after mechanical reperfusion requires further investigation.

## Figures and Tables

**Table 1:** Differential expression of parenchymal hematoma-related microRNAs was identified in the rat stroke model and oxygen-glucose deprivation/reoxygenation models of neuron, astrocyte, microglia, brain microvascular endothelial cell, and pericyte using quantitative RT-PCR.

	MicroRNAs	In Vivo Stroke Model		In-Vitro OGD/R Model				
		Peri-Infarct	Infarction Core	Neuron	Astrocyte	Microglia	BMEC	Pericyte
1	miR-1-3p	NS	NS	-	-	-	-	-
2	miR-25-5p	NS	NS	-	-	-	-	-
3	miR-27b-3p	NS	NS	-	-	-	-	-
4	miR-29a-5p	Down	Down	NS	Down	NS	NS	NS
5	miR-29c-3p	Down	Down	Up	Down	Up	Down	Up
6	miR-30a-3p	NS	NS	-	-	-	-	-
7	miR-126a-5p	Down	Down	NS	Down	Up	Down	NS
8	miR-132-3p	Down	Down	Down	Down	Down	Up	Down
9	miR-136-3p	Down	Down	Down	NS	Down	NS	Up
10	miR-138-5p	NS	NS	-	-	-	-	-
11	miR-139-5p	NS	Down	-	-	-	-	-
12	miR-142-3p	Down	Down	NS	Down	NS	Down	NS
13	miR-153-5p	Down	Down	Down	Down	NS	Up	Up
14	miR-195-3p	Up	Up	Down	NS	NS	Up	Up
15	miR-218a-5p	Down	Down	Down	Down	Down	Up	Down
16	miR-219a-2-3p	Down	Down	Up	Down	NS	NS	NS
17	miR-320-5p	NS	NS	-	-	-	-	-
18	miR-337-3p	NS	Down	-	-	-	-	-
19	miR-369-5p	Down	Down	Down	Down	NS	NS	NS
20	miR-376a-5p	Down	Down	Down	NS	NS	NS	NS
21	miR-376b-5p	Down	Down	Down	NS	Up	NS	Up
22	miR-381-3p	NS	Up	-	-	-	-	-
23	miR-383-5p	Down	Down	Down	Down	NS	NS	NS
24	miR-487b-5p	NS	NS	-	-	-	-	-
25	miR-582-3p	NS	Down	-	-	-	-	-

Differential expression of parenchymal hematoma-related microRNAs was identified in the rat stroke model and oxygen-glucose deprivation/reoxygenation (OGD/R) models of neuron, astrocyte, microglia, brain microvascular endothelial cell (BMEC), and pericyte by quantitative RT-PCR. The down-regulated and up-regulated expression of microRNAs were compared between the parenchymal hematoma group or OGD/R group at 24 h and the control group. Results were expressed as Mean ± SD. Statistical comparisons were done with the Student *t*-test and one-way ANOVA. NS, not significant.

## Data Availability

All data supporting the findings of this study are available in the paper, as well as supplementary information.

## Supplementary Materials

The following supporting information can be downloaded at: https://www.mdpi.com/article/10.3390/biomedicines13020449/s1, Figure S1: Fourteen parenchymal hematoma-related microRNAs were assessed by quantitative RT-PCR in the peri-infarct and infarction core in rats with hemorrhagic infarction; Figure S2: Dynamic expression of 14 microRNAs in neurons after oxygen-glucose deprivation/reoxygenation at 0, 6, 12, 24, 48, 72 hours; Figure S3: Dynamic expression of 14 microRNAs in astrocytes after oxygen-glucose deprivation/reoxygenation at 0, 3, 6, 12, 24 hours; Figure S4: Dynamic expression of 14 microRNAs in microglia after oxygen-glucose deprivation/reoxygenation at 0, 3, 6, 12, 24 hours; Figure S5: Dynamic expression of 14 microRNAs in brain microvascular endothelial cells after oxygen-glucose deprivation/reoxygenation at 0, 3, 6, 12, 24 hours; Figure S6: Dynamic expression of 14 microRNAs in pericytes after oxygen-glucose deprivation/reoxygenation at 0, 3, 6, 12, 24 hours; Figure S7: The microRNA-mRNA regulatory axes of parenchymal hematoma with involved signaling pathways; Figure S8: Dynamic expression of interleukin 1 beta and toll-like receptor 4 in the oxygen-glucose deprivation reoxygenation model of neuron, astrocyte, microglia, and pericyte; Figure S9: Dynamic expression of cAMP responsive element binding protein 1, forkhead box O1, and signal transducer and activator of transcription 1 in the oxygen-glucose deprivation reoxygenation model of brain microvascular endothelial cell and pericyte; Table S1: Sequences of the primer for mature microRNAs used in the qRT-PCR; Table S2: Sequences of the primer for key predicted target mRNAs used in the qRT-PCR; Table S3: The microRNA-mRNA regulatory axes of parenchymal hematoma based on the prediction tool and PCR examination.
