# Supplementary material for: MicroRNAs Associated with Parenchymal Hematoma After Endovascular Mechanical Reperfusion for Acute Ischemic Stroke in Rats
Source: Biomedicines. 2025 Feb 12;13(2):449. doi: 10.3390/biomedicines13020449 (PMC11853160; doi:10.3390/biomedicines13020449)
Supplement: Supplementary file 1 [file biomedicines-13-00449-s001.zip › biomedicines-3423943-supplementary.pdf]

biomedicines

MDPI

Supplementary Table S1. Sequences of the primer for mature rat microRNAs used in the qRT-PCR.

Primer name	Primer sequence	Length
rno-miR-1-3p	Forward:5'GGGGCTGGAATGTAAAGAAGT3'<br>Reverse: 5'GTGCGTGTCGTGGAGTCG3'	65
rno-miR-25-5p	Forward:5'TTTTGGCGGAGACACGG3'<br>Reverse: 5'GTGCGTGTCGTGGAGTCG3'	63
rno-miR-27b-3p	Forward:5'GGGGGTTCACAGTGGCTAAG3'<br>Reverse: 5'GTGCGTGTCGTGGAGTCG3'	64
rno-miR-29a-5p	Forward:5'GGGACTGATTTCTTTTGGT3'<br>Reverse: 5'GTGCGTGTCGTGGAGTCG3'	65
rno-miR-29c-3p	Forward:5'GGGTAGCACCATTTGAAA3'<br>Reverse: 5'GTGCGTGTCGTGGAGTCG3'	63
rno-miR-30a-3p	Forward:5'GGGCTTTCAGTCGGATGTT3'<br>Reverse: 5'GTGCGTGTCGTGGAGTCG3'	63
rno-miR-126a-5p	Forward:5'GGGGGCATTATTACTTTTGG3'<br>Reverse: 5'GTGCGTGTCGTGGAGTCG3'	64
rno-miR-132-3p	Forward:5'GGGGTAACAGTCTACAGCC3'<br>Reverse: 5'CAGTGCGTGTCGTGGAG3'	66
rno-miR-136-3p	Forward:5'GGGGACATCATCGTCTCAAAT3'<br>Reverse: 5'GTGCGTGTCGTGGAGTCG3'	65
rno-miR-138-5p	Forward:5'GGGGCTGGTGTTGTGAATC3'<br>Reverse: 5'GTGCGTGTCGTGGAGTCG3'	63
rno-miR-139-5p	Forward:5'GGGGTTCTACAGTGCACGTGT3'<br>Reverse: 5'GTGCGTGTCGTGGAGTCG3'	65
rno-miR-142-3p	Forward:5'ACCCCCTGTAGTGTTTCCTACTT3'<br>Reverse: 5'GTGCGTGTCGTGGAGTCG3'	67
rno-miR-153-5p	Forward:5'GGGGGTCATTTTTGTGATGTT3'<br>Reverse: 5'GTGCGTGTCGTGGAGTCG3'	65
rno-miR-195-3p	Forward:5'GCTTCGGCAGCACATATACTAAAAT3'<br>Reverse: 5'CGCTTCACGAATTTGCGTGTCAT3'	63
rno-miR-218a-5p	Forward:5'GGGGTTGTGCTTGATCTAA3'<br>Reverse: 5'CAGTGCGTGTCGTGGAG3'	65
rno-miR-219a-2-3p	Forward:5'AGGGAGAATTGTGGCTGGA3'<br>Reverse: 5'GTGCGTGTCGTGGAGTCG3'	64
rno-miR-320-5p	Forward:5'ACGCCTTCTCTTCCCGGT3'<br>Reverse: 5'GTGCGTGTCGTGGAGTCG3'	62
rno-miR-337-3p	Forward:5'GGGGGTTCAGCTCCTATATGAT3'<br>Reverse: 5'GTGCGTGTCGTGGAGTCG3'	66
rno-miR-369-5p	Forward:5'GGGGAGATCGACCGTGTTAT3'<br>Reverse: 5'GTGCGTGTCGTGGAGTCG3'	64
rno-miR-376a-5p	Forward:5'GGGGGGGTAGATTCTCCTTC3'<br>Reverse: 5'GTGCGTGTCGTGGAGTCG3'	64
rno-miR-376b-5p	Forward:5'GGGGGGTGGATATTCCTTCTA3'<br>Reverse: 5'GTGCGTGTCGTGGAGTCG3'	65
rno-miR-381-3p	Forward:5'AGGGGGGTATACAAGGGCA3'<br>Reverse: 5'GTGCGTGTCGTGGAGTCG3'	63
rno-miR-383-5p	Forward:5'GGGGGACAGATCAGAAGGTG3'<br>Reverse: 5'GTGCGTGTCGTGGAGTCG3'	65
rno-miR-487b-5p	Forward:5'CCCGAGTGGTTATCCCTGTC3'<br>Reverse: 5'GTGCGTGTCGTGGAGTCG3'	65
rno-miR-582-3p	Forward:5'GGGGGAACCTGTTGAACAA3'	65

	Reverse: 5'GTGCGTGTCGTGGAGTCG3'	
U6	Forward:5'GCTTCGGCAGCACATATACTAAAAT3'<br>Reverse: 5'CGCTTCACGAATTTGCGTGTCAT3'	89

Supplementary Table S2. Sequences of the primer for key predicted target mRNAs used in the qRT-PCR.

Primer name	Primer sequence	Length
Actb	Forward:5'CAGCAAGCAGGAGTACGATGA3'<br>Reverse:5'GTAACAGTCCGCCTAGAAGCAT3'	70
Agt	Forward:5'CAGAGCCAACCTTTGAGCCT3'<br>Reverse:5'CCGATCCTCAGCCTCTAGCTT3'	113
Bcl2	Forward:5'ACGAGTGGGATACTGGAGATGA3'<br>Reverse:5'TCTCAGGCTGGAAGGAGAAGAT3'	81
Bcl2l1	Forward:5'GAGAGAGGCTGGCGATGAGT3'<br>Reverse:5'CAAAGCTCTGATATGCTGTCCCT3'	101
Casp3	Forward:5'GCGGTATTGAGACAGACAGTGG3'<br>Reverse:5'TGCGGTAGAGTAAGCATACAGGA3'	92
Ccl5	Forward:5'GTGCCAACCCAGAGAAGAAGT3'<br>Reverse:5'AGAGCAAGCAATGACAGGAAAG3'	114
Ccnd1	Forward:5'GGGACCGTGATGGCATTAC3'<br>Reverse:5'AAAGCCTCCTGTGTGAAAGACTT3'	70
Cd40	Forward:5'CCAGATGATTGGGTCGGTG3'<br>Reverse:5'ACAAAATCCTCGTGAAGGCTT3'	90
Cd44	Forward:5'CCAGCCAGTGACAGGTTCCA3'<br>Reverse:5'CATGATGTGCAGGATGAAACCA3'	97
Cd80	Forward:5'CAAGTGTCCAAGTCGGTGAGAG3'<br>Reverse:5'TTTTTGCCAGTAGATTCGGTGT3'	96
Creb1	Forward:5'GCTAAAAGCACTTAAGGACCTTTAC3'<br>Reverse:5'CCAGTCCATTTTCCACCACAAT3'	87
Csf1	Forward:5'GTGAAAGTTTGCCTCGGTGCT3'<br>Reverse:5'CGCTTCCTTGCTCGCTAGTG3'	113
Csf1r	Forward:5'CCTCGCAGGTTTGGGATGAC3'<br>Reverse:5'GCTCTGAAGGATCACTCTGTGGA3'	74
Csf2	Forward:5'ATTGTGGTCTACAGTTTCTCAGCA3'<br>Reverse:5'CCAGAGCACGCATGTCATTTA3'	118
Ctnnb1	Forward:5'AAGTTCTTGGCTATTACGACAGACT3'<br>Reverse:5'AGCTTCTCGTACGTGTAGGTTCTC3'	134
Cxcl1	Forward:5'TTGTGCAGTTTAAAGATGGTAGGC3'<br>Reverse:5'GACTCTCATCTCTCCGCCCTTC3'	125
Cxcl10	Forward:5'CACATCCCGAGCCAACCTT3'<br>Reverse:5'TCCCTTGAGTCCCACTCAGACT3'	94
Cxcl12	Forward:5'TTCACTCTCCGTCCACCTCG3'<br>Reverse:5'GGCTGACTGGCTTACCGTCA3'	150
Cxcr4	Forward:5'ATCTGTGACCGCCTTTACCC3'<br>Reverse:5'CAGGACAGGATGACGATGCC3'	101
Esr1	Forward:5'ATCAAGCACACTAAGAAGAATAGCC3'<br>Reverse:5'CCATCATTGAGGCTTCACTGAA3'	139
Fos	Forward:5'CCTGAAGAGGAAGAGAAACGG3'<br>Reverse:5'AATCTCGGTCTGCAACGCA3'	150
Foxo1	Forward:5'GCCCAGCACCCACAGGTTAT3'<br>Reverse:5'ACTTGAAAGCACGCCAGGA3'	131

Foxp3	Forward:5'ATCCCTCTCTGTCAGTCCACTTC3'<br>Reverse:5'TGCAGTGTGTCCGGCTGTACT3'	127
Hif1a	Forward:5'TCTGGCAATGTCTCCATTACCT3'<br>Reverse:5'CAGTGACTCTGGGCTTGACTCTA3'	115
Ido1	Forward:5'ACAGCAATGCCTCACAGTCAA3'<br>Reverse:5'AGCAAAGCCCACATCTTCATC3'	90
Ifnb1	Forward:5'TTGCCATTCAAGTGATGCTCC3'<br>Reverse:5'CAACAATAGTCTCATTCCACCCAG3'	84
Igf1	Forward:5'CATCTCTTCTACCTGGCACTCTG3'<br>Reverse:5'TTGGTCCACACACGAACTGAA3'	115
Il13	Forward:5'CACAGGACCCAGAGGATATTGA3'<br>Reverse:5'GCGGAAAAGTTGCTTGGAGTAA3'	135
Il15	Forward:5'GCAATGAACTGCTTTCTCCTGG3'<br>Reverse:5'TGCAAGGTAGAGCACGTTTCTT3'	96
Il17a	Forward:5'GCTACTGAACCTGGAGGCTACA3'<br>Reverse:5'CTCGGCGTTTGGACACACT3'	73
Il1a	Forward:5'CCTCGTCCTAAGTCACTCGCA3'<br>Reverse:5'GTATCATATGTCGGGCTGGTTC3'	115
Il1b	Forward:5'TGGGATGATGACGACCTGCT3'<br>Reverse:5'TGGCTTATGTTCTGTCCATTGAG3'	143
Il2	Forward:5'AGCAACTGTGGTGGAATTTCTG3'<br>Reverse:5'CCTTGTGTGTTATAAGTAGGAGGCA3'	103
Jun	Forward:5'GTCCAGCAATGGGCACATC3'<br>Reverse:5'GGCAGCGTATTCTGGCTATG3'	138
Lep	Forward:5'TTTCAGGGCACATTAGCATCC3'<br>Reverse:5'TCTTCTAGTCCCGAGCACTTTG3'	122
Mtor	Forward:5'GCAGATTTGCCAACTACCTTC3'<br>Reverse:5'CACTTCAAACTCCACATACTCAG3'	134
Pparg	Forward:5'GGAATCAGCTCTGTGGACCTCTC3'<br>Reverse:5'TGGAGAAATCAACCGTGGTAAAG3'	85
Pten	Forward:5'CCTCAGAAAAAGTGGAAAATGG3'<br>Reverse:5'TTATCCGCACGCTCTATACTACAA3'	76
Ptgs2	Forward:5'CTACCATCTGGCTTCGGGA3'<br>Reverse:5'GGTTTGGAACAGTCGCTCGT3'	89
Ptprc	Forward:5'AGATTGCCGATGAGGGTAGAC3'<br>Reverse:5'CTTTGATGGGAAACTTGCTGAA3'	78
Sirt1	Forward:5'AGGGAACCTCTGCCTCATCTA3'<br>Reverse:5'GGCATACTCGCCACCTAACC3'	99
Stat1	Forward:5'CTTTATCAGCAAGGAGCGAGAAC3'<br>Reverse:5'CAACAGCATGGAAGTCGGGT3'	155
Tlr2	Forward:5'TTTTCAGCCTATGCCCGAC3'<br>Reverse:5'CCTGTATCCCTGTACTGGACAAGT3'	77
Tlr4	Forward:5'TCCACAAGAGCCGGAAAGTT3'<br>Reverse:5'TGAAGATGATGCCAGAGCGG3'	126
Tp53	Forward:5'GAGTTGTTAGAAGGCCCAGAGG3'<br>Reverse:5'AGAAGGGACGGAAGATGACAG3'	132
Vcam1	Forward:5'AACGCTCGCTCAGATTGGA3'<br>Reverse:5'TTCACCTTCCCATTTAGTGGACT3'	114
β-actin	Forward:5'CGAGTACAACCTTCTTGCAGC3'<br>Reverse:5'ACCCATACCCACCATCACAC3'	202

Actb, actin, beta; Agt, angiotensinogen; Bcl2, B-cell lymphoma 2; Bcl2l1, Bcl2-like 1; Casp3, caspase 3; Ccl5, C-C motif chemokine ligand 5; Ccnd1, cyclin D1; Cd40, CD40 molecule; Cd44, CD44

molecule; Cd80, CD80 molecule; Creb1, cAMP responsive element binding protein 1; Csf1, colony stimulating factor 1; Csf1r, colony stimulating factor 1 receptor; Csf2, colony stimulating factor 2; Ctnnb1, catenin beta 1; Cxcl1, C-X-C motif chemokine ligand 1; Cxcl10, C-X-C motif chemokine ligand 10; Cxcl12, C-X-C motif chemokine ligand 12; Cxcr4, C-X-C motif chemokine receptor 4; Esr1, estrogen receptor 1; Fos, Fos proto-oncogene, AP-1 transcription factor subunit; Foxo1, forkhead box O1; Foxp3, forkhead box P3; Hif1a, hypoxia inducible factor 1 subunit alpha; Ido1, indoleamine 2,3-dioxygenase 1; Ifnb1, interferon beta 1; Igf1, insulin-like growth factor 1; Il13, interleukin 13; Il15, interleukin 15; Il17a, interleukin 17A; Il1a, interleukin 1 alpha; Il1b, interleukin 1 beta; Il2, interleukin 2; Jun, Jun proto-oncogene, AP-1 transcription factor subunit; Lep, leptin; Mtor, mechanistic target of rapamycin kinase; Pparg, peroxisome proliferator-activated receptor gamma; Pten, phosphatase and tensin homolog; Ptgs2, prostaglandin-endoperoxide synthase 2; Ptprc, protein tyrosine phosphatase, receptor type, C; Sirt1, sirtuin 1; Stat1, signal transducer and activator of transcription 1; Tlr2, toll-like receptor 2; Tlr4, toll-like receptor 4; Tp53, tumor protein p53; Vcam1, vascular cell adhesion molecule 1.

Supplementary Table S3. The microRNA-mRNA regulatory axes of parenchymal hematoma based on the prediction tool and PCR examination.

	microRNA	microRNA expression	Target gene symbol	Gene expression	Mechanism of target gene	Top ten enriched pathways
1	miRNA-126a-5p	Down	Cd44	Up	Immune, inflammation, apoptosis	Proteoglycans in cancer
			Cd80	Up	Immune, inflammation	
			Csf1	Up	Immune, inflammation	Ras signaling pathway, Rap1 signaling pathway, MAPK signaling pathway
			Csf2	Up	Immune, inflammation, apoptosis	
			Cxcl10	Up	Immune, inflammation, oxidative stress	
			Esr1	Up	Immune, oxidative stress, apoptosis	Proteoglycans in cancer, Breast cancer
			Ido1	Up	Immune, inflammation	
			Igf1	Up	Inflammation, oxidative stress, apoptosis	Ras signaling pathway, Rap1 signaling pathway, Proteoglycans in cancer, Signaling pathways regulating pluripotency of stem cells, MAPK signaling pathway, FoxO signaling pathway, Breast cancer, Growth hormone synthesis, secretion and action
			Il1a	Up	Immune, inflammation, oxidative stress, apoptosis	MAPK signaling pathway
			Jun	Up	Immune, inflammation, oxidative stress, apoptosis	MAPK signaling pathway, Breast cancer, Wnt signaling pathway
			Ptgs2	Up	Immune, inflammation, oxidative stress, apoptosis	
			Tlr4	Up	Immune, inflammation, oxidative stress, apoptosis	Proteoglycans in cancer
2	miRNA-136-3p	Down	Cxcl1	Up	Immune, apoptosis	
			Hif1a	Up	Immune, inflammation, oxidative stress, apoptosis	Proteoglycans in cancer
			Igf1	Up	Inflammation, oxidative stress, apoptosis	Ras signaling pathway, Rap1 signaling pathway, Proteoglycans in cancer, Signaling pathways regulating pluripotency of stem cells, MAPK signaling pathway, FoxO signaling pathway, Breast cancer, Growth hormone synthesis, secretion and action
3	miRNA-153-5p	Down	Casp3	Up	Oxidative stress, apoptosis	Proteoglycans in cancer, MAPK signaling pathway
			Cd44	Up	Immune, inflammation, apoptosis	Proteoglycans in cancer
			Cd80	Up	Immune, inflammation	
			Esr1	Up	Immune, oxidative stress, apoptosis	Proteoglycans in cancer, Breast cancer

			Hif1a	Up	Immune, inflammation, oxidative stress, apoptosis	Proteoglycans in cancer
			Ido1	Up	Immune, inflammation,	
			Igf1	Up	Inflammation, oxidative stress, apoptosis	Ras signaling pathway, Rap1 signaling pathway, Proteoglycans in cancer, Signaling pathways regulating pluripotency of stem cells, MAPK signaling pathway, FoxO signaling pathway, Breast cancer, Growth hormone synthesis, secretion and action
			Il1a	Up	Immune, inflammation, oxidative stress, apoptosis	MAPK signaling pathway
			Il1b	Up	Immune, inflammation, oxidative stress, apoptosis	MAPK signaling pathway
			Ptgs2	Up	Immune, inflammation, oxidative stress, apoptosis	
			Ptprc	Up	Immune	
			Tlr4	Up	Immune, inflammation, oxidative stress, apoptosis	Proteoglycans in cancer
4	miRNA-218a-5p	Down	Il1b	Up	Immune, inflammation, oxidative stress, apoptosis	MAPK signaling pathway
			Tlr4	Up	Immune, inflammation, oxidative stress, apoptosis	Proteoglycans in cancer
5	miRNA-29a-5p	Down	Casp3	Up	Oxidative stress, apoptosis	Proteoglycans in cancer, MAPK signaling pathway
			Ccl5	Up	Immune, inflammation, oxidative stress, apoptosis	
			Cd44	Up	Immune, inflammation, apoptosis	Proteoglycans in cancer
			Ido1	Up	Immune, inflammation	
			Ptgs2	Up	Immune, inflammation, oxidative stress, apoptosis	
			Tlr4	Up	Immune, inflammation, oxidative stress, apoptosis	Proteoglycans in cancer
6	miRNA-29c-3p	Down	Igf1	Up	Inflammation, oxidative stress, apoptosis	Ras signaling pathway, Rap1 signaling pathway, Proteoglycans in cancer, Signaling pathways regulating pluripotency of stem cells, MAPK signaling pathway, FoxO signaling pathway, Breast cancer, Growth hormone synthesis, secretion and action
7	miRNA-376a-5p	Down	Cxcl1	Up	Immune, apoptosis	
			Esr1	Up	Immune, oxidative stress, apoptosis	Proteoglycans in cancer, Breast cancer
			Ifnb1	Up	Immune, apoptosis	
			Igf1	Up	Inflammation, oxidative stress, apoptosis	Ras signaling pathway, Rap1 signaling pathway, Proteoglycans in cancer, Signaling pathways regulating pluripotency of stem cells, MAPK signaling pathway, FoxO signaling pathway, Breast cancer, Growth hormone synthesis, secretion and action
			Tlr2	Up	Immune, apoptosis	Proteoglycans in cancer
8	miRNA-376b-5p	Down	Fos	Up	Immune, inflammation, oxidative stress, apoptosis	MAPK signaling pathway, Breast cancer, Growth hormone synthesis, secretion and action
			Hif1a	Up	Immune, inflammation, oxidative stress, apoptosis	Proteoglycans in cancer
			Igf1	Up	Inflammation, oxidative stress, apoptosis	Ras signaling pathway, Rap1 signaling pathway, Proteoglycans in cancer, Signaling pathways regulating pluripotency of stem cells, MAPK signaling pathway, FoxO signaling pathway, Breast cancer, Growth hormone synthesis, secretion and action

			Ptgs2	Up	Immune, inflammation, oxidative stress, apoptosis	
			Tlr4	Up	Immune, inflammation, oxidative stress, apoptosis	Proteoglycans in cancer
9	miRNA-195-3p	Up	Creb1	Down	Immune, inflammation, oxidative stress, apoptosis	Growth hormone synthesis, secretion and action
			Foxo1	Down	Oxidative stress, apoptosis	FoxO signaling pathway
			Stat1	Down	Immune, inflammation, oxidative stress, apoptosis	Growth hormone synthesis, secretion and action

Casp3, caspase 3; Ccl5, C-C motif chemokine ligand 5; Cd44, CD44 molecule; Cd80, CD80 molecule; Creb1, cAMP responsive element binding protein 1; Csf1, colony stimulating factor 1; Csf2, colony stimulating factor 2; Cxcl1, C-X-C motif chemokine ligand 1; Cxcl10, C-X-C motif chemokine ligand 10; Esr1, estrogen receptor 1; Fos, Fos proto-oncogene, AP-1 transcription factor subunit; Foxo1, forkhead box O1; Hif1a, hypoxia inducible factor 1 subunit alpha; Ido1, indoleamine 2,3-dioxygenase 1; Ifnb1, interferon beta 1; Igf1, insulin-like growth factor 1; Il1a, interleukin 1 alpha; Il1b, interleukin 1 beta; Jun, Jun proto-oncogene, AP-1 transcription factor subunit; Ptgs2, prostaglandin-endoperoxide synthase 2; Ptprc, protein tyrosine phosphatase, receptor type, C; Stat1, signal transducer and activator of transcription 1; Tlr2, toll-like receptor 2; Tlr4, toll-like receptor 4; MiRNA, microRNA.

Supplementary Figure S1. Fourteen parenchymal hematoma-related microRNAs were assessed by quantitative RT-PCR in the peri-infarct and infarction core in rats with hemorrhagic infarction.

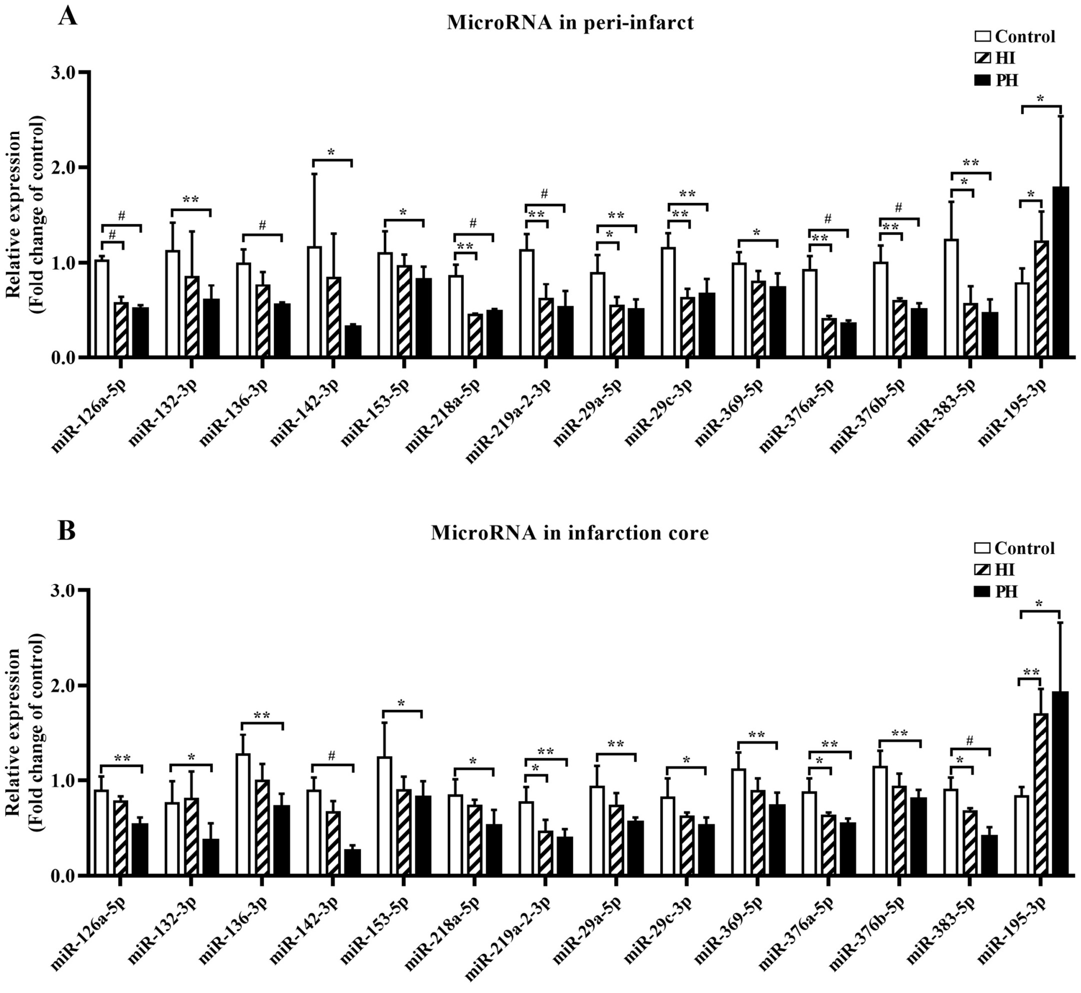

Ten of the 14 parenchymal hematoma (PH)-related microRNAs did not show a significant difference in expression between the peri-infarct or infarction core in rats with hemorrhagic infarction (HI) (n=3) and those in the sham-operated group (n=5). The remaining PH-related microRNAs showed

a trend towards greater differential expression in the PH group (n=5) compared to rats with hemorrhagic infarction. A, microRNAs in the peri-infarct. B, microRNAs in the infarction core. Results were expressed as Mean ± SD. Statistical comparisons were done with Student t-test. *P<0.05; **P<0.01; #P<0.001.

Supplementary Figure S2. Dynamic expression of 14 microRNAs in rat neurons after oxygen-glucose deprivation (OGD) reoxygenation at 0, 6, 12, 24, 48, 72 hours.

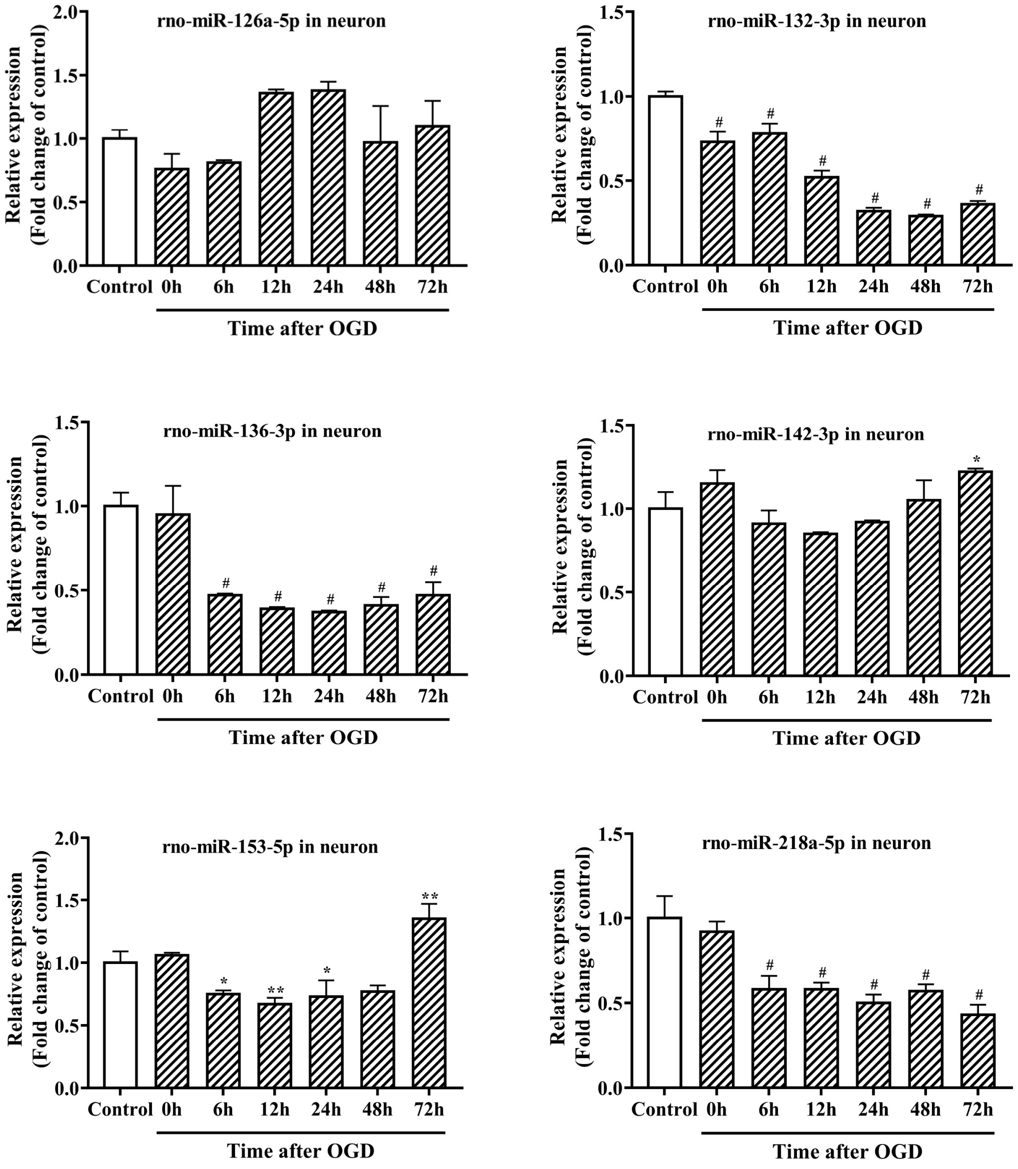

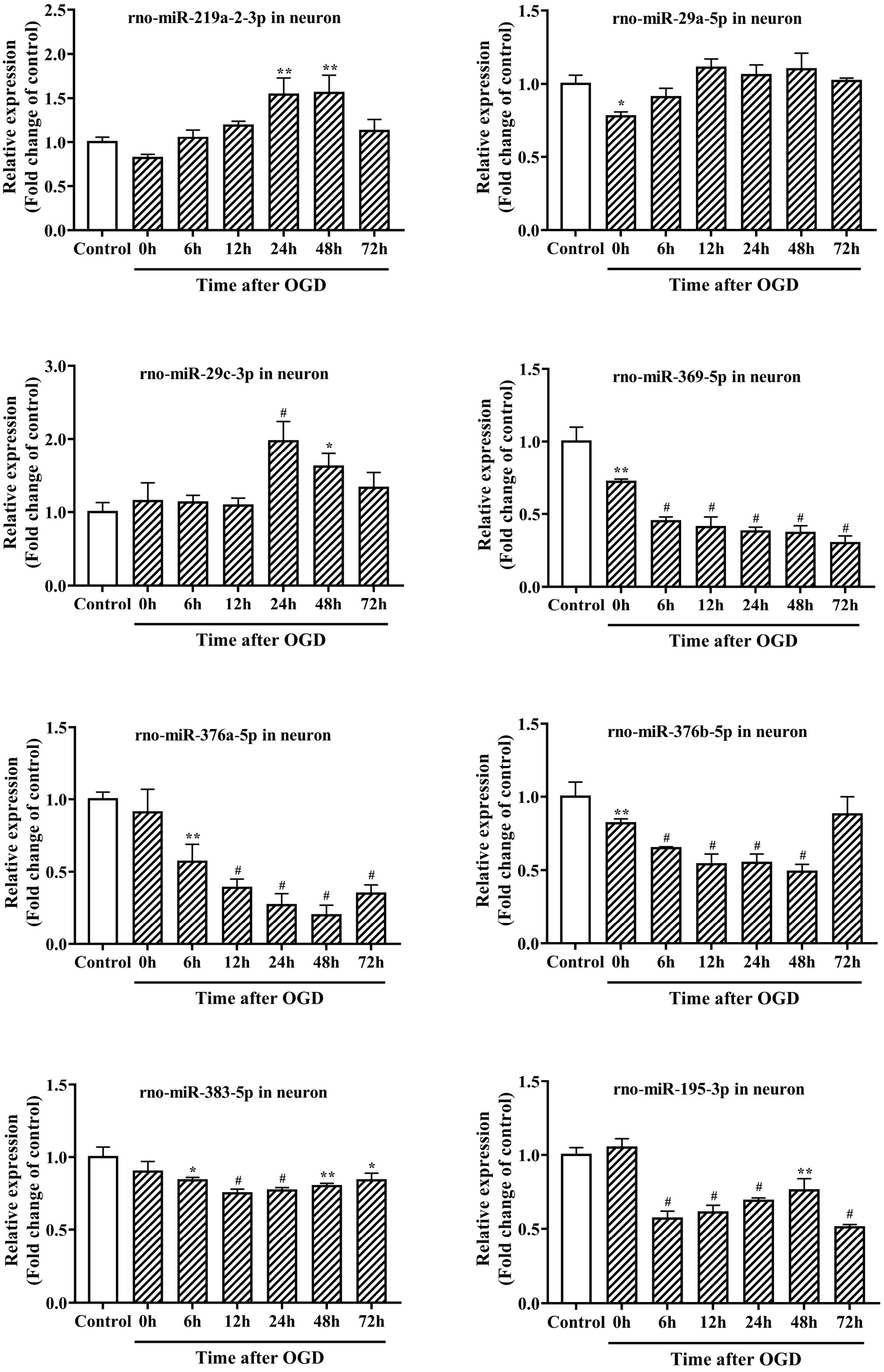

Results were expressed as Mean ± SD from three independent experiments. Statistical comparisons were done with one-way ANOVA for the groups of different reoxygenation times. *P<0.05; **P<0.01; #P<0.001.

Supplementary Figure S3. Dynamic expression of 14 microRNAs in rat astrocytes after oxygen-glucose deprivation (OGD) reoxygenation at 0, 3, 6, 12, 24 hours.

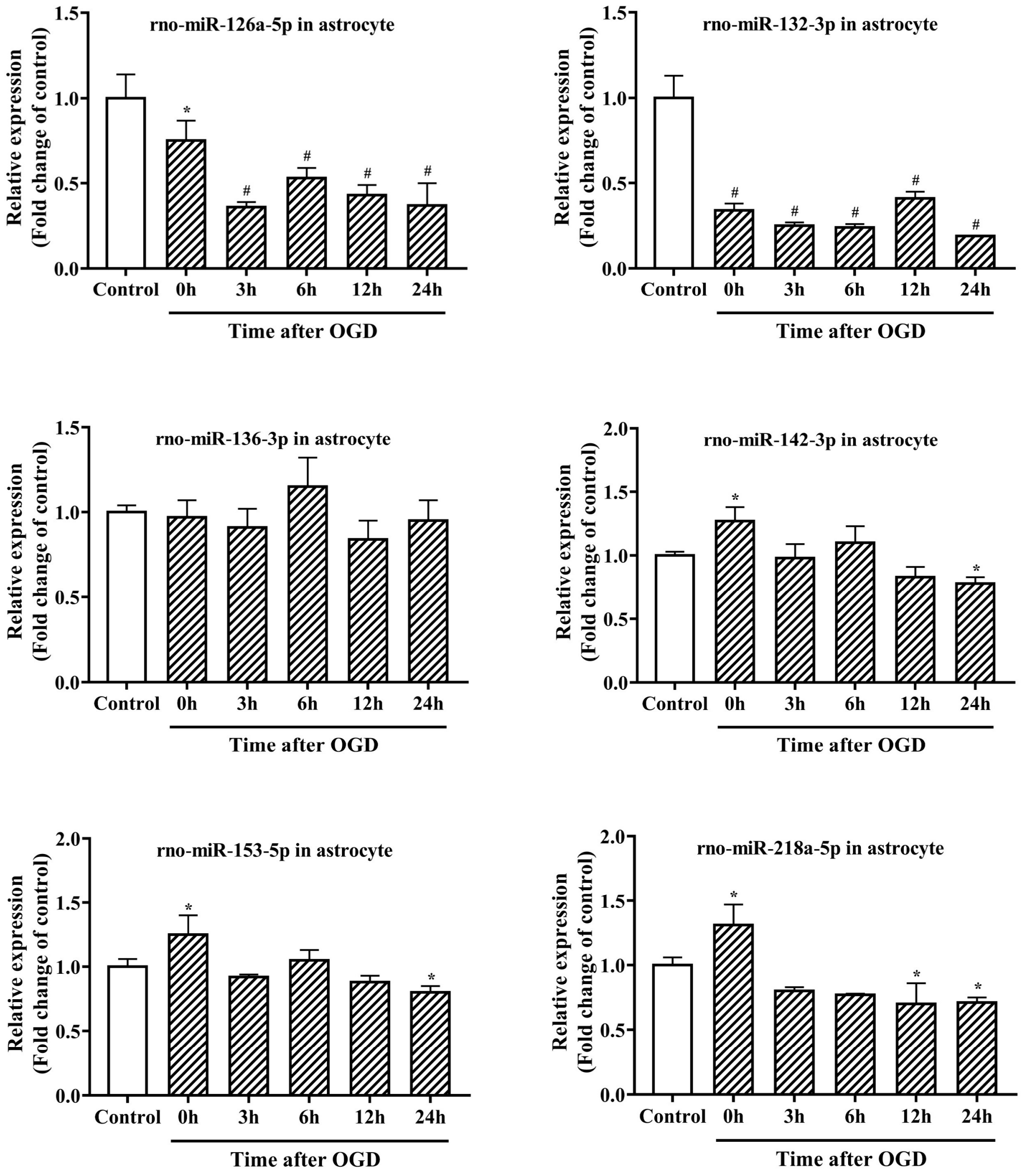
rno-miR-126a-5p in astrocyte
rno-miR-132-3p in astrocyte
rno-miR-136-3p in astrocyte
rno-miR-142-3p in astrocyte
rno-miR-153-5p in astrocyte
rno-miR-218a-5p in astrocyte
Relative expression
(Fold change of control)
Control
0h
3h
6h
12h
24h
Time after OGD
0.0
0.5
1.0
1.5
2.0
*
#

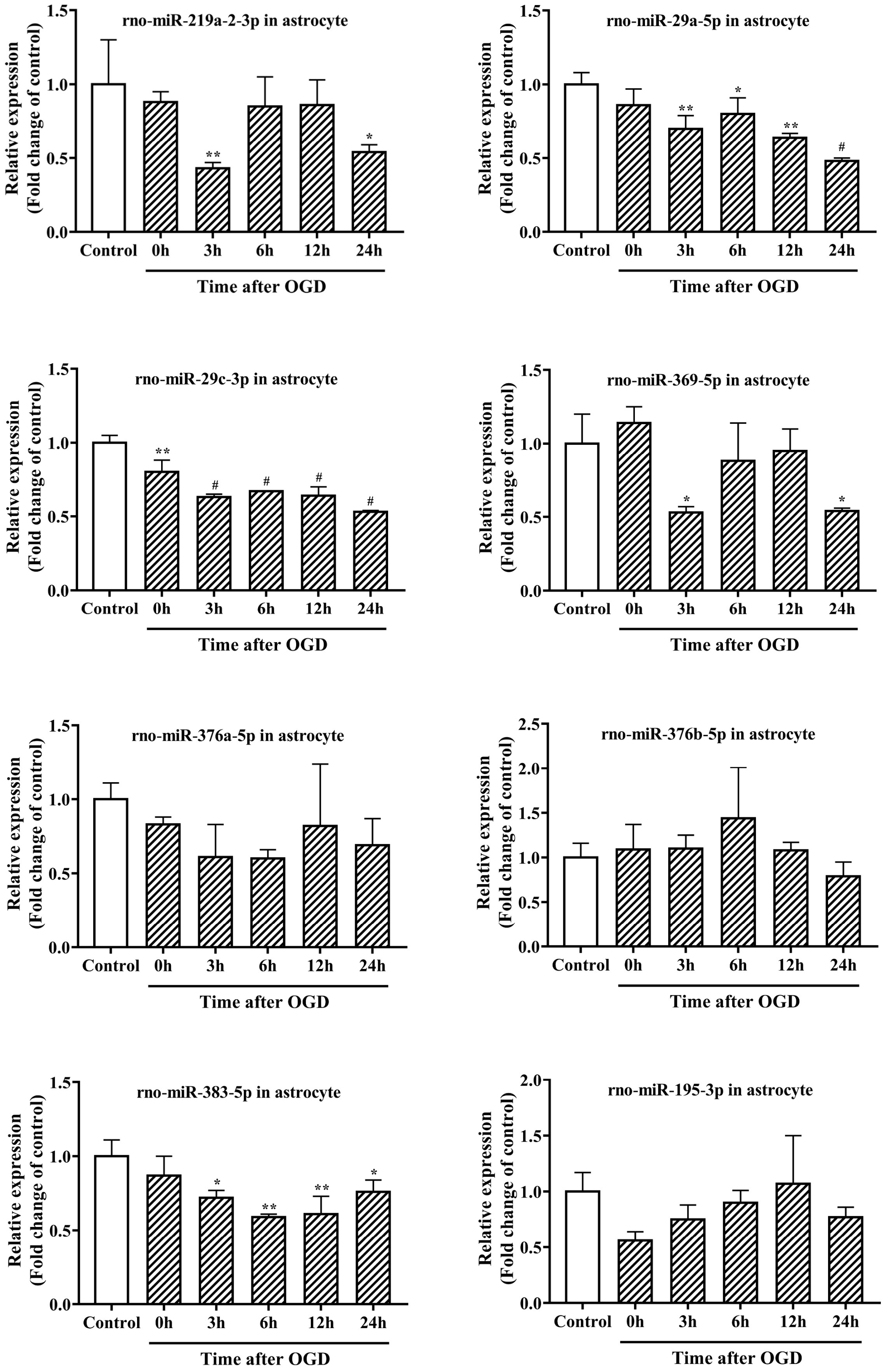

Results were expressed as Mean ± SD from three independent experiments. Statistical comparisons were done with one-way ANOVA for the groups of different reoxygenation times. *P<0.05; **P<0.01; #P<0.001.

Supplementary Figure S4. Dynamic expression of 14 microRNAs in rat microglia after oxygen-glucose deprivation (OGD) reoxygenation at 0, 3, 6, 12, 24 hours.

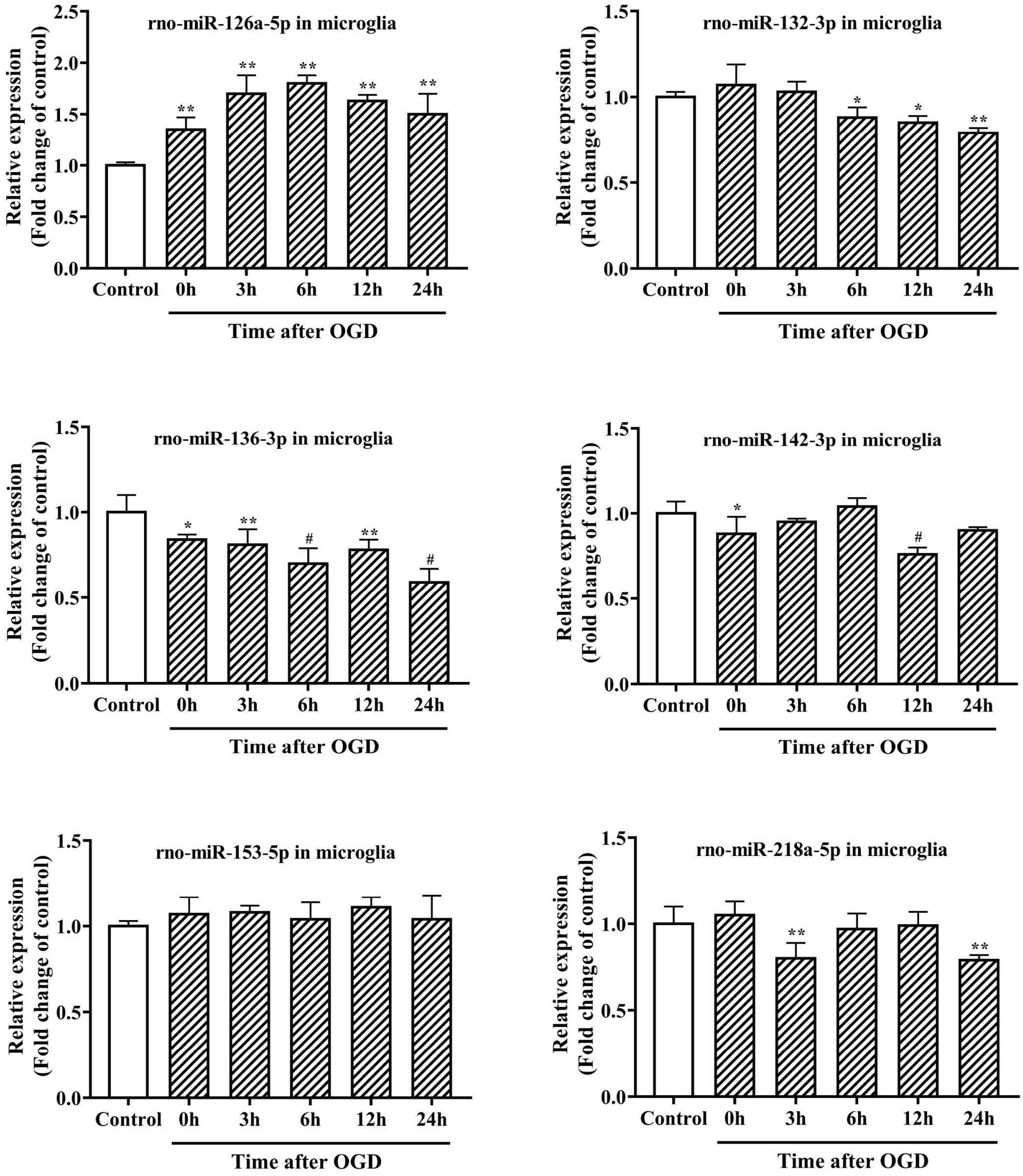
rno-miR-126a-5p in microglia
Relative expression (Fold change of control)
0.0
0.5
1.0
1.5
2.0
2.5
**
**
**
**
**
Control
0h
3h
6h
12h
24h
Time after OGD
rno-miR-132-3p in microglia
Relative expression (Fold change of control)
0.0
0.5
1.0
1.5
*
*
**
Control
0h
3h
6h
12h
24h
Time after OGD
rno-miR-136-3p in microglia
Relative expression (Fold change of control)
0.0
0.5
1.0
1.5
*
**
#
**
#
Control
0h
3h
6h
12h
24h
Time after OGD
rno-miR-142-3p in microglia
Relative expression (Fold change of control)
0.0
0.5
1.0
1.5
*
#
Control
0h
3h
6h
12h
24h
Time after OGD
rno-miR-153-5p in microglia
Relative expression (Fold change of control)
0.0
0.5
1.0
1.5
Control
0h
3h
6h
12h
24h
Time after OGD
rno-miR-218a-5p in microglia
Relative expression (Fold change of control)
0.0
0.5
1.0
1.5
**
**
Control
0h
3h
6h
12h
24h
Time after OGD

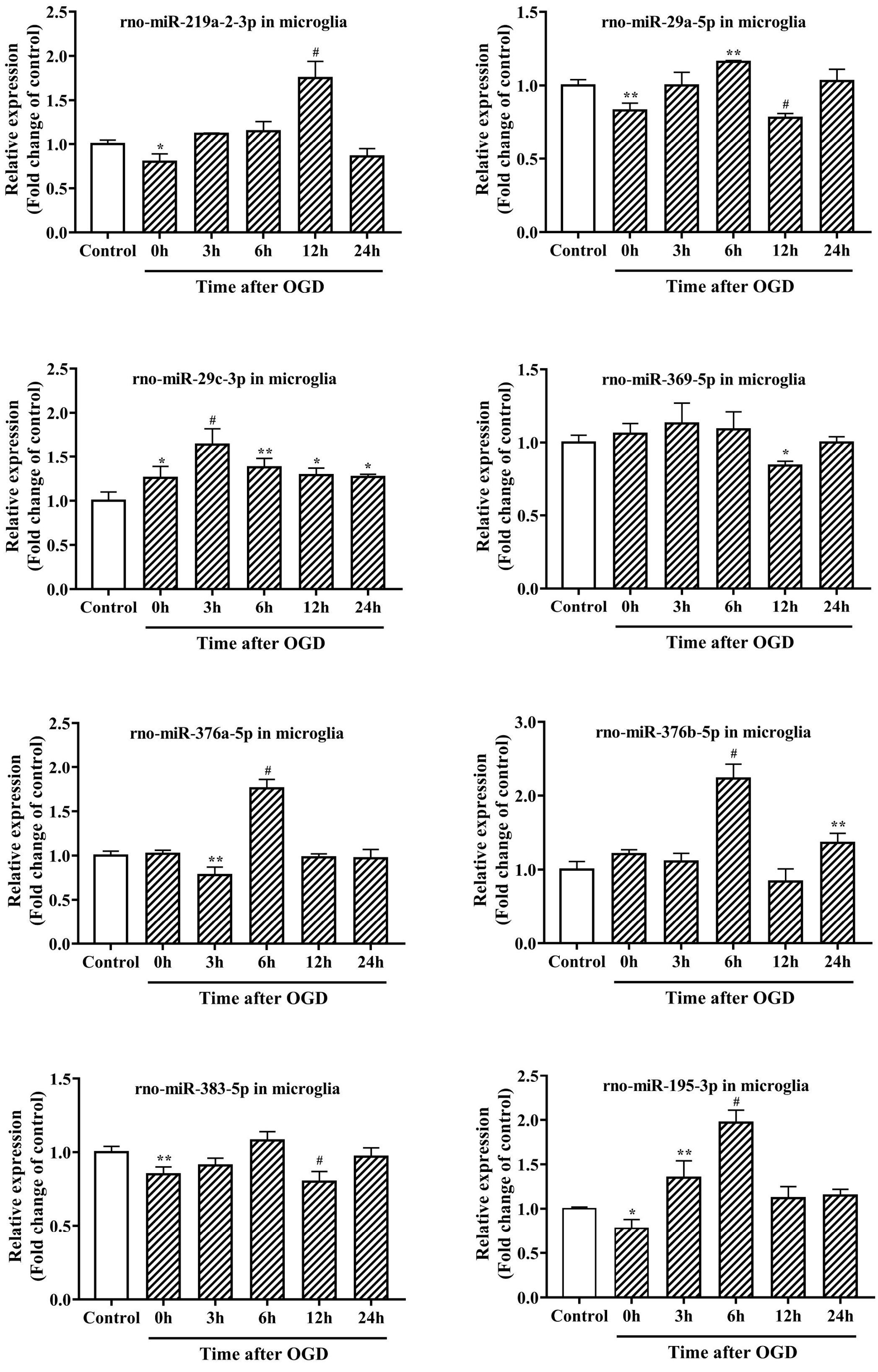

Results were expressed as Mean ± SD from three independent experiments. Statistical comparisons were done with one-way ANOVA for the groups of different reoxygenation times. *P<0.05; **P<0.01; #P<0.001.

Supplementary Figure S5. Dynamic expression of 14 microRNAs in rat brain microvascular endothelial cell (BMEC) after oxygen-glucose deprivation (OGD) reoxygenation at 0, 3, 6, 12, 24 hours.

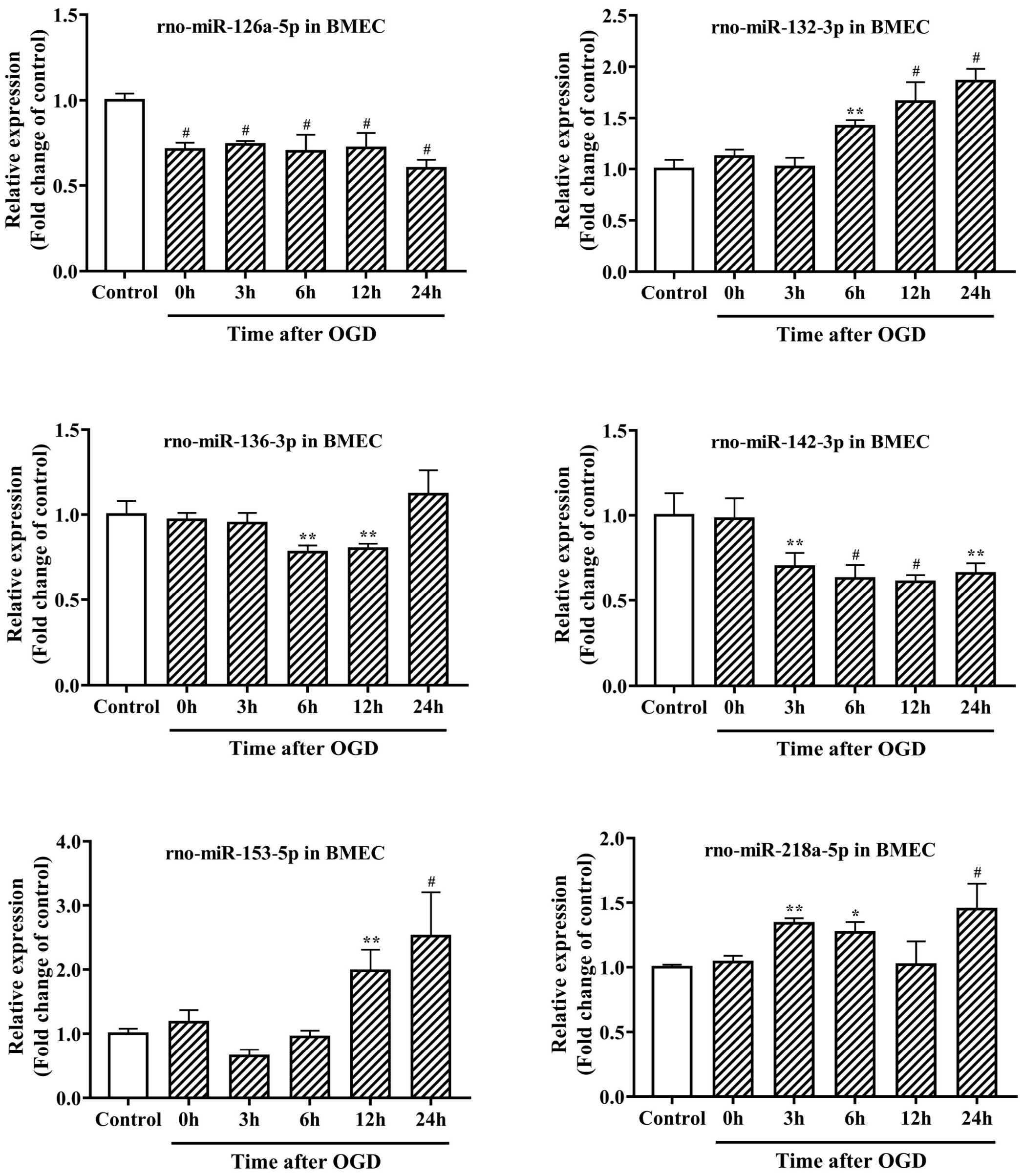
rno-miR-126a-5p in BMEC
Relative expression (Fold change of control)
1.5
1.0
0.5
0.0
#
Control
0h
3h
6h
12h
24h
Time after OGD
rno-miR-132-3p in BMEC
Relative expression (Fold change of control)
2.5
2.0
1.5
1.0
0.5
0.0
**
#
Control
0h
3h
6h
12h
24h
Time after OGD
rno-miR-136-3p in BMEC
Relative expression (Fold change of control)
1.5
1.0
0.5
0.0
**
Control
0h
3h
6h
12h
24h
Time after OGD
rno-miR-142-3p in BMEC
Relative expression (Fold change of control)
1.5
1.0
0.5
0.0
**
#
Control
0h
3h
6h
12h
24h
Time after OGD
rno-miR-153-5p in BMEC
Relative expression (Fold change of control)
4.0
3.0
2.0
1.0
0.0
**
#
Control
0h
3h
6h
12h
24h
Time after OGD
rno-miR-218a-5p in BMEC
Relative expression (Fold change of control)
2.0
1.5
1.0
0.5
0.0
**
*
#
Control
0h
3h
6h
12h
24h
Time after OGD

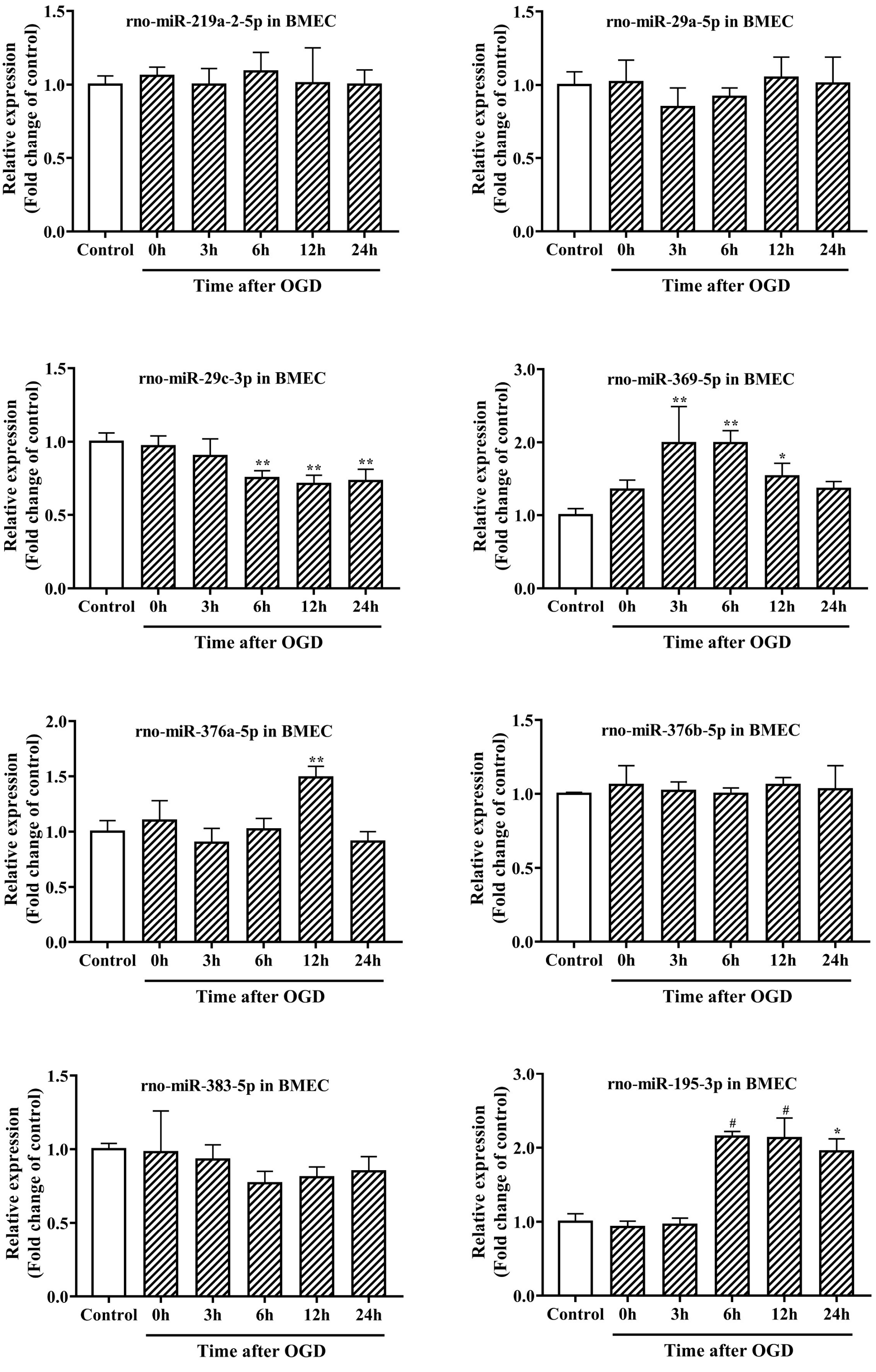

Results were expressed as Mean ± SD from three independent experiments. Statistical comparisons were done with one-way ANOVA for the groups of different reoxygenation times. *P<0.05; **P<0.01; #P<0.001.

Supplementary Figure S6. Dynamic expression of 14 microRNAs in rat pericytes after oxygen-glucose deprivation (OGD) reoxygenation at 0, 3, 6, 12, 24 hours.

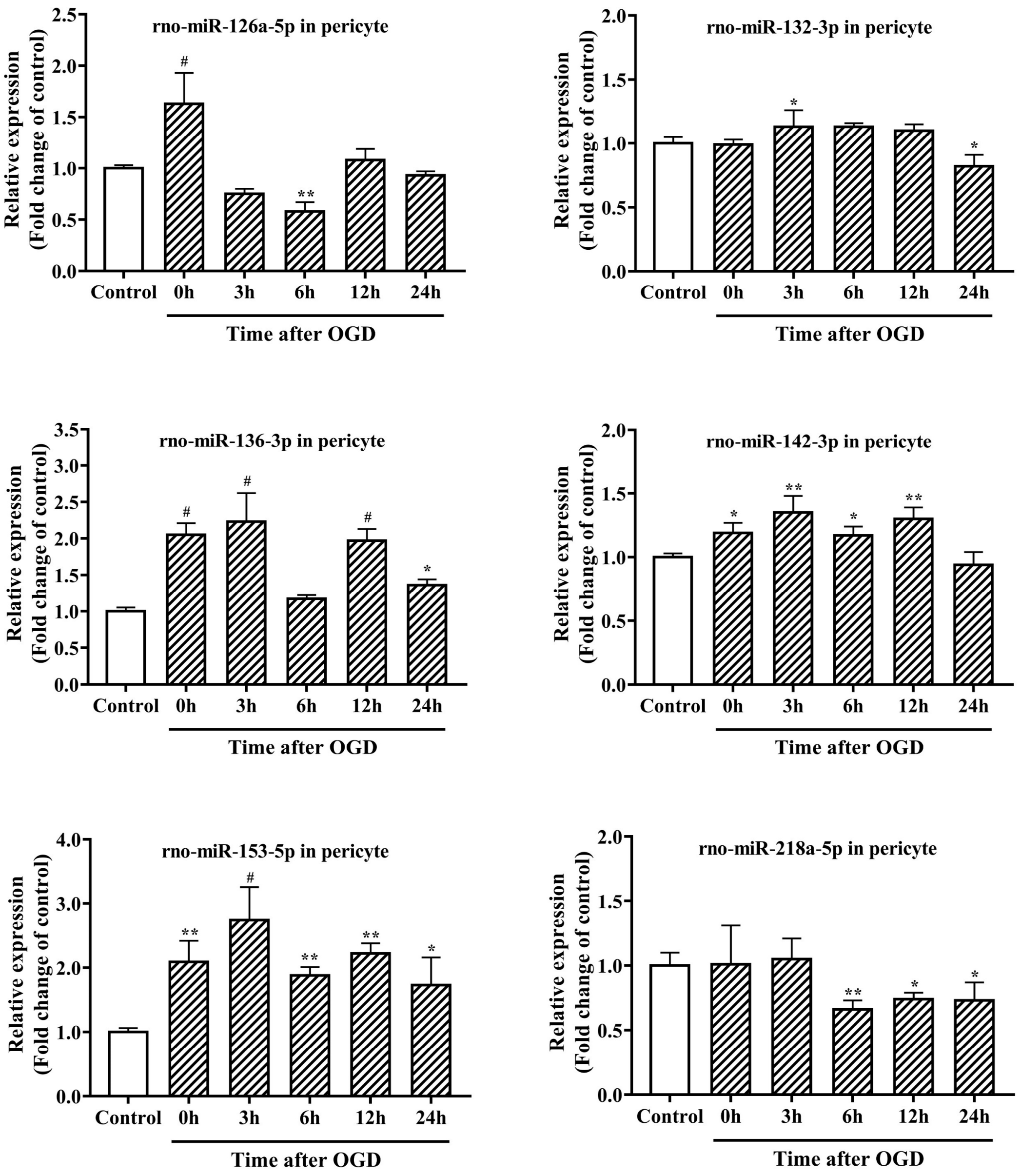

rno-miR-126a-5p in pericyte
Relative expression (Fold change of control)
0.0
0.5
1.0
1.5
2.0
2.5
#
**
Control
0h
3h
6h
12h
24h
Time after OGD
rno-miR-132-3p in pericyte
Relative expression (Fold change of control)
0.0
0.5
1.0
1.5
2.0
*
*
Control
0h
3h
6h
12h
24h
Time after OGD
rno-miR-136-3p in pericyte
Relative expression (Fold change of control)
0.0
0.5
1.0
1.5
2.0
2.5
3.0
3.5
#
#
#
*
Control
0h
3h
6h
12h
24h
Time after OGD
rno-miR-142-3p in pericyte
Relative expression (Fold change of control)
0.0
0.5
1.0
1.5
2.0
*
**
*
**
Control
0h
3h
6h
12h
24h
Time after OGD
rno-miR-153-5p in pericyte
Relative expression (Fold change of control)
0.0
1.0
2.0
3.0
4.0
**
#
**
**
*
Control
0h
3h
6h
12h
24h
Time after OGD
rno-miR-218a-5p in pericyte
Relative expression (Fold change of control)
0.0
0.5
1.0
1.5
2.0
**
*
*
Control
0h
3h
6h
12h
24h
Time after OGD

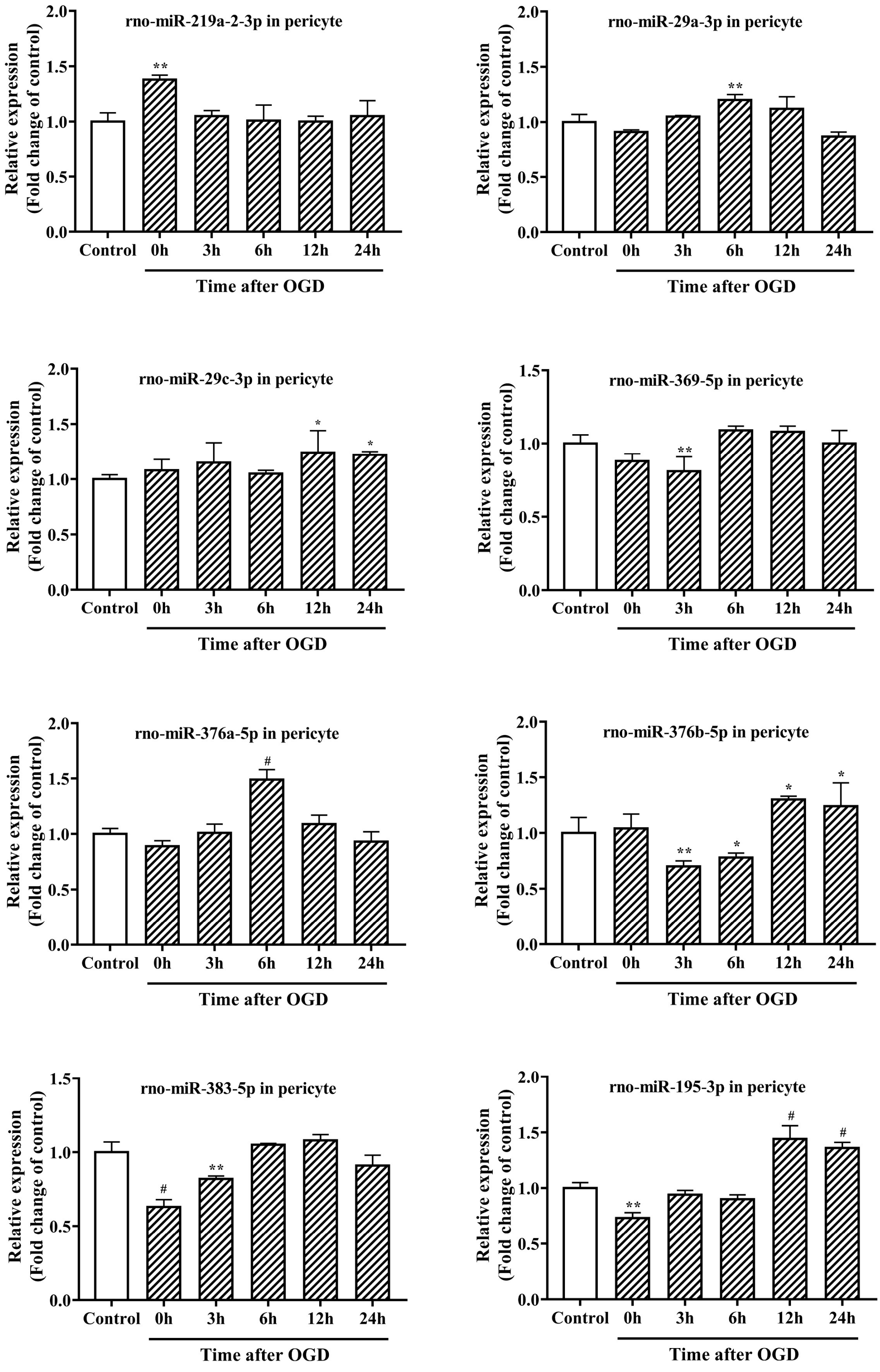

Results were expressed as Mean ± SD from three independent experiments. Statistical comparisons were done with one-way ANOVA for the groups of different reoxygenation times. *P<0.05; **P<0.01; #P<0.001.

Supplementary Figure S7. The microRNA-mRNA regulatory axes of parenchymal hematoma with involved signaling pathways.

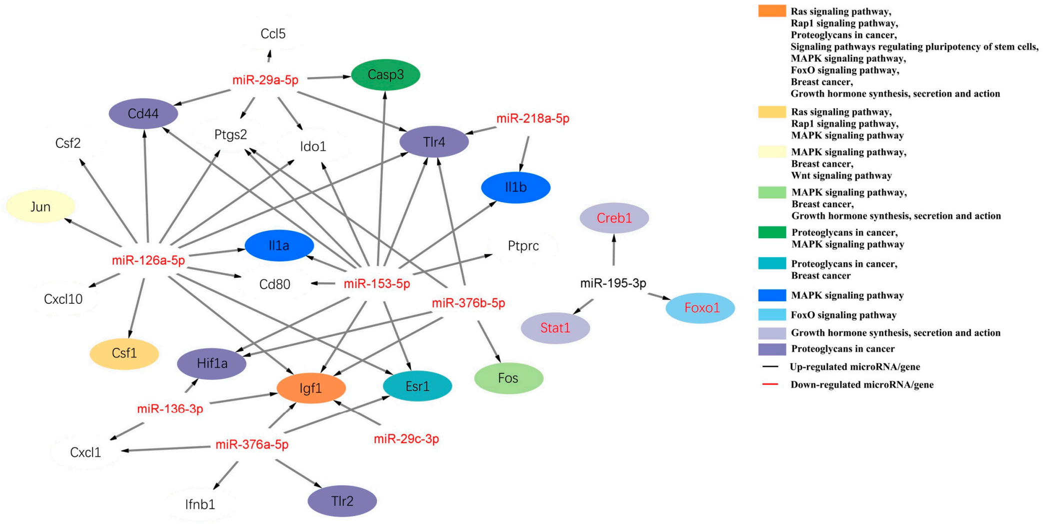

The microRNA-mRNA regulatory axes of parenchymal hematoma based on the prediction tool and PCR examination, including nine microRNAs (eight down-regulated microRNAs and one up-regulated microRNA) and their 24 essential target genes with involved key signaling pathways. These signaling pathways included MAPK signaling pathway, Ras signaling pathway, Rap1 signaling pathway, proteoglycans in cancer, Wnt signaling pathway, signaling pathways regulating pluripotency of stem cells, breast cancer, FoxO signaling pathway, growth hormone synthesis, secretion and action.

Supplementary Figure S8. Dynamic expression of interleukin 1 beta and toll-like receptor 4 in the oxygen-glucose deprivation (OGD) reoxygenation model of neuron, astrocyte, microglia, and pericyte.

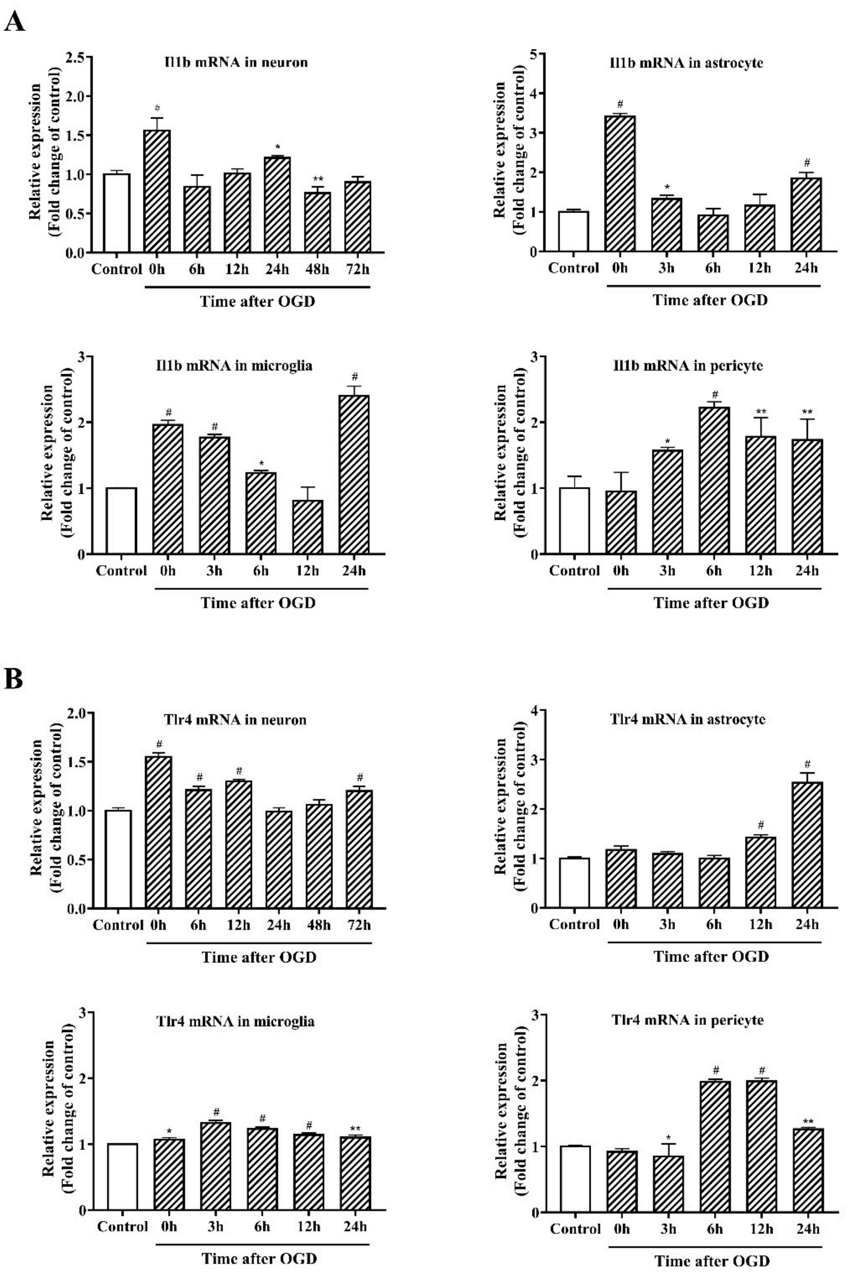

The expression of interleukin 1 beta (Il1b) and toll-like receptor 4 (Tlr4) as the predicted target gene of microRNA-218a-5p increased in the oxygen-glucose deprivation (OGD) reoxygenation model of neuron, astrocyte, microglia, and pericyte. (A) Il1b. (B) Tlr4. Results were expressed as Mean ± SD from three independent experiments. Statistical comparisons were done with one-way ANOVA. *P<0.05; **P<0.01; #P<0.001.

Supplementary Figure S9. Dynamic expression of cAMP responsive element binding protein 1, forkhead box O1, and signal transducer and activator of transcription 1 in the oxygen-glucose deprivation (OGD) reoxygenation model of brain microvascular endothelial cell and pericyte.

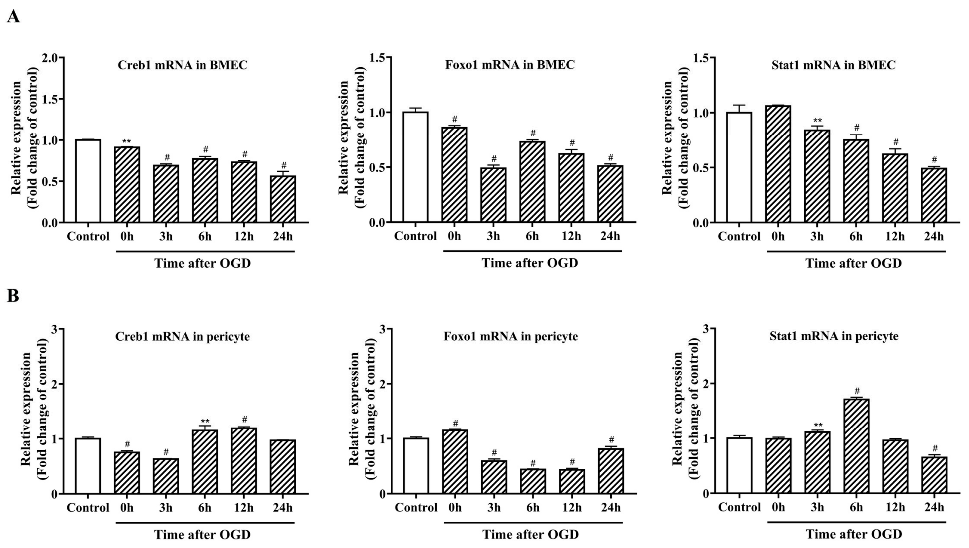

The expression of cAMP responsive element binding protein 1 (Creb1), forkhead box O1 (Foxo1), and signal transducer and activator of transcription 1 (Stat1) as the predicted target gene of microRNA-195-3p was shown in the oxygen-glucose deprivation (OGD) reoxygenation model of brain microvascular endothelial cell (BMEC) and pericyte. (A) genes in the model of BMEC. (B) genes in the model of pericyte. Results were expressed as Mean ± SD from three independent experiments. Statistical comparisons were done with one-way ANOVA. **P<0.01; #P<0.001.
